# Rewiring Aromatic Compound Consumption: Chromosomal Amplification and Evolution of a Foreign Pathway in *Acinetobacter baylyi* ADP1

**DOI:** 10.1021/acssynbio.5c00341

**Published:** 2025-08-27

**Authors:** Alyssa C. Baugh, Melissa P. Tumen-Velasquez, Isabella R. Zempel, Chantel V. Duscent-Maitland, Lauren E. Slarks, Justin B. Defalco, Christopher W. Johnson, Gregg T. Beckham, Ellen L. Neidle

**Affiliations:** † Department of Microbiology, 1355 University of Georgia, Athens, Georgia 30602, United States; ‡ Renewable Resources and Enabling Sciences Center, 53405 National Renewable Energy Laboratory, Golden, Colorado 80401, United States

**Keywords:** protocatechuate, Acinetobacter baylyi, gene amplification, aromatic compounds

## Abstract

Rational engineering strategies that seek to harness the remarkable diversity of microbial metabolism can be limited by incomplete biological knowledge. As described here, a novel approach to address this challenge involved replacing a native pathway for degrading lignin-derived aromatic compounds via *ortho* cleavage of protocatechuate in *Acinetobacter baylyi* ADP1 with a foreign *meta*-cleavage pathway that uses different enzymes, metabolites, and redox carriers. This alteration may improve lignin valorization and coordinate catabolism with bioproduction strategies. When a 14-kbp region of foreign DNA was inserted in the chromosome, the heterologous genes failed to confer growth on target substrates. Regional gene dosage was increased using a synthetic DNA fragment to promote recombination, and higher copy number enabled growth. During adaptive laboratory evolution, compensatory mutations arose that permit growth with one copy of the foreign genes. This complex metabolic remodeling was accomplished without assumptions about the impediments that initially prevented growth. To understand the changes that emerged, a novel transformation assay identified a combination of mutations sufficient for the new phenotype. Three unexpected changes were revealed: loss of one foreign enzyme, loss of one native enzyme, and loss of a two-component transcriptional regulatory system. This study establishes that large multicopy tandem arrays of poorly adapted pathway genes can confer new functions and improve understanding of metabolism.

## Graphical abstract

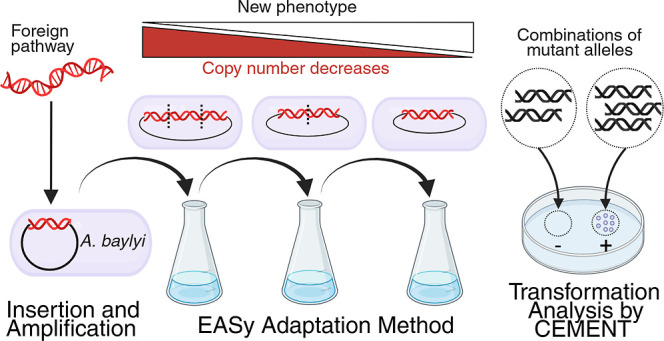

## Introduction

Promising strategies for lignin valorization use microbes to funnel aromatic compounds into valuable products.^1–3^ These metabolic approaches circumvent some of the problems caused by the chemical complexity and heterogeneity of lignin-derived feedstocks.^4,5^ While synthetic biology and adaptive laboratory evolution are powerful techniques to modify microorganisms for this purpose, success depends on the capabilities and genetic tractability of the host. The aromatic compound degradation capabilities of *Acinetobacter baylyi* ADP1 are among the best of a set of bacteria tested for their ability to depolymerize lignin and catabolize the resulting mixture of low molecular weight aromatic compounds.^5^ These native activities can be expanded by using metabolic engineering to express foreign and synthetic pathways, an approach that may advance the goal of biological lignin valorization.^1–3^ Moreover, the potential of *A. baylyi* for bioproduction continues to be developed.^6–8^

*A. baylyi* has exceptionally high rates of natural transformation and recombination.^9^ Its unique genetic system enables EASy (Evolution by Amplification and Synthetic biology), a method that promotes variation in the copy number of a genomic segment. Variation in gene dosage can result in new metabolic abilities, including novel substrate consumption by allowing changes in expression to evolve.^10–12^ In this study, we sought to develop a generalizable method for the introduction and optimization of multistep pathways to effect substantial metabolic change. Many factors can account for the suboptimal performance of genes in a new host. Often, efforts to establish functionality involve alternative promoters, modified ribosome binding sites, codon optimization, enzyme engineering and/or mutagenesis. However, such modification becomes increasingly difficult with complex pathways. Targeted gene amplification, coupled with adaptive evolution, provides a promising engineering strategy that requires no knowledge of constraints that limit functionality.

In nature, adaptation to new environments can be mediated by fluctuation in gene dosage following stochastic duplication events.^13^ At a population-level scale, this variability can underlie the emergence of enzyme variants, new metabolic capabilities, new regulation, and mutations that increase the fitness of poorly adapted pathways.^14,15^ Weak side activities of enzymes can confer metabolic novelty at an organismal level, as shown experimentally and computationally in *Escherichia coli*.^15,16^ To harness such adaptation in the laboratory, we can use the natural transformability and ease of introducing and selecting chromosomal duplications in *A. baylyi*. In previous studies, a 10-kbp segment of native DNA was moved to multiple genomic loci, and spontaneous mutants were selected that all carry tandem duplications of segments 20–300 kbp in size.^17^ With copy number ranging from two to more than 100, many mutants had more than 1 Mbp of amplified DNA on a chromosome that is normally 3.6-Mbp.^18^ These results suggest the feasibility of introducing and amplifying large DNA segments to confer new metabolic pathways. Furthermore, the induction of a precise duplication, using the EASy method, obviates the need to await rare spontaneous duplication events.

Our goal here was to replace a natural pathway for the degradation of 4-hydroxybenzoate (4HB), and other aromatic compounds that are degraded through protocatechuate (PCA), with a non-native pathway.^19^ The enzymes that cleave PCA in the native and non-native pathways open the aromatic ring at different positions, generating different products. The subsequent catabolic routes vary with respect to their intermediates, their cofactors, carbon efficiency, and the products generated for entry into the tricarboxylic acid (TCA) cycle (Figure 1). There are several reasons for engineering this change. First, bioproduction strategies to synthesize industrially important chemicals can be designed to exploit differences between the native pathway (the PCA branch of the *ortho*-cleaving β-ketoadipate pathway) and the foreign pathway (a 2,3-*meta*-cleavage pathway). For example, unlike the *ortho*-cleavage pathway (encoded by *pca* genes native to *A. baylyi*), the *meta*-cleavage pathway (encoded by the *pra* genes from *Paenibacillus* sp. JJ-1b) directly generates pyruvate, a precursor that can be used to produce valuable compounds such as acetoin, l-alanine, and citramalate.^20,21^ Furthermore, differences in the reducing equivalents generated and the amount of CO_2_ emitted can substantially influence the product yield in a predictable fashion, as we previously demonstrated.^22^ The regeneration of NADH by the foreign pathway (Figure 1B), which does not occur in the native pathway, may be advantageous for the bioproduction of some compounds listed above.

**Figure 1:**
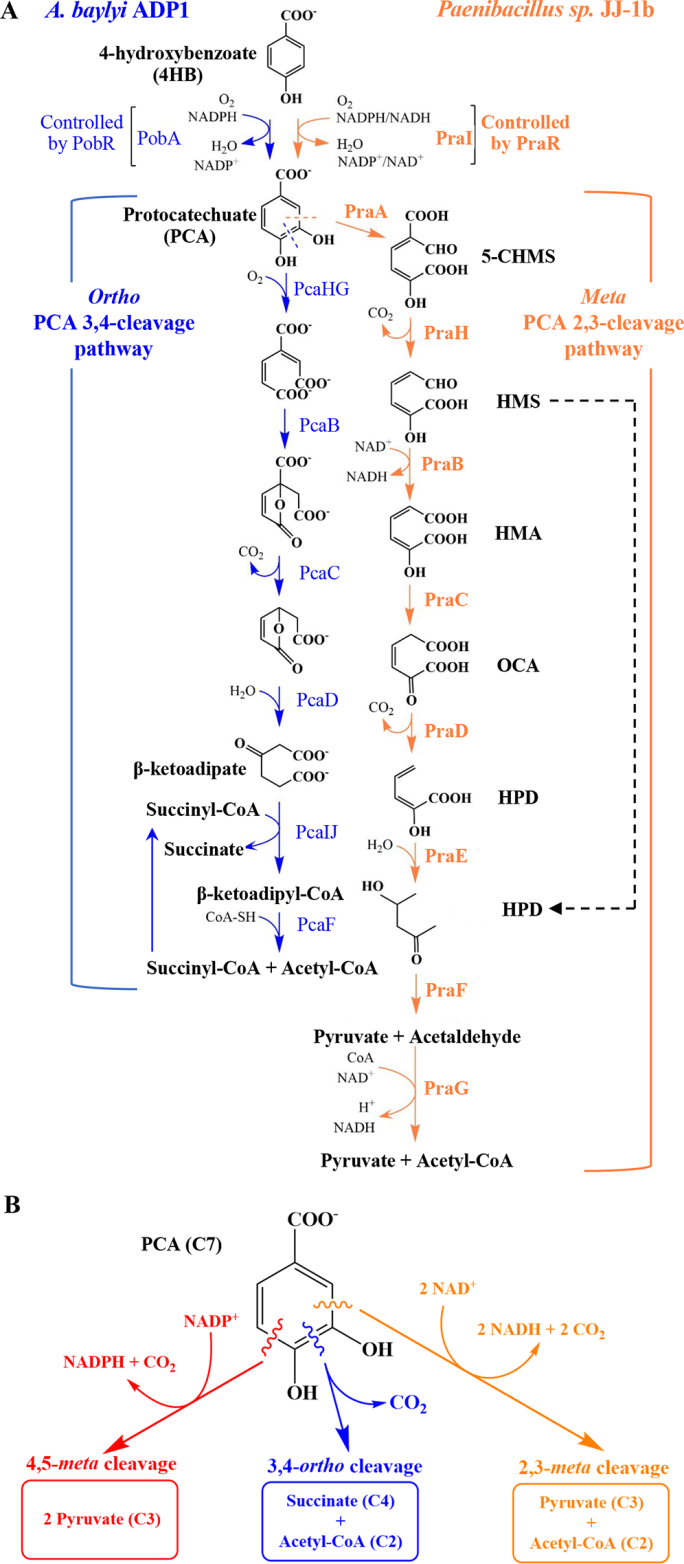
4HB catabolism. (A) The native route in *A. baylyi* ADP1 involves hydroxylation of 4HB to produce PCA, followed by ring cleavage by PCA 3,4-dioxygenase (PcaHG). The scission between both hydroxyl groups is denoted as *ortho* cleavage. This pathway (blue), encoded by *pca* genes, is one branch of the β-ketoadipate pathway. Transcription is regulated by PcaU.^28^ A foreign pathway from *Paenibacillus* sp. JJ-1b (orange) involves *meta* cleavage (adjacent to both hydroxyl groups) by PCA 2,3-dioxygenase (PraA).^29^ This pathway is encoded by *pra* genes. Transcription is regulated by PraR. The dashed black arrow indicates a hydrolase-catalyzed reaction encoded by *xylF* on TOL plasmid pWW0.^30^ (B) Some microbes encode a PCA 4,5-dioxygenase and associated catabolic pathway (red).^19^ These three PCA catabolic pathways use different electron carriers and oxidize the 7-carbon aromatic substrate (C7) to different products, reflecting altered carbon distribution. Abbreviations: 5-CHMS (5-carboxy-2-hydroxymuconate-6-semialdehyde), HMS (2-hydroxymuconate-6-semialdehyde), HMA (2-hydroxymuconate), OCA (4-oxalocrotonate), HPD (2-hydroxypenta-2,3-dienoate), HOV (4-hydroxy-2-oxovalerate).

Another reason for engineering this pathway exchange in *A. baylyi* relates to broadening aromatic compound catabolism. Because this bacterium shows promise for lignin valorization, it may be possible to increase its cometabolism of diverse compounds in lignin-derived mixtures. Branching pathways to increase the consumption of target compounds could be introduced as modules that connect to metabolites uniquely formed in the *meta*-cleavage pathway. For example, the metabolism of syringol might be engineered through conversion to pyrogallol followed by cleavage to 2-hydroxymuconate (HMA), a metabolite in the non-native pathway.^23,24^ In addition, the generation of ADP1-derived strains with different degradative capabilities and pathways can contribute to new strategies for using *A. baylyi* consortia for lignin valorization.^25^

Engineering the exchange of the *pra*- and *pca*-encoded pathways was designed to advance methodology needed to realize the potential of *A. baylyi* as a platform organism. Previously, EASy was used to extend the β-ketoadipate pathway by one or two catabolic steps without fundamentally changing this pathway.^10–12^ Here, introduction of an entire foreign pathway was expected to cause wide-ranging perturbations; the metabolites are atypical, toxic metabolites may accumulate, and adverse interactions may occur with native proteins. Redox balance may be disturbed. Unlike methods that depend on a fixed expression level, the expansion and contraction of a chromosomal array allows a beneficial expression level to be selected.^15^ This dynamic interplay between chromosomal mutations and gene dosage mimics natural evolution.^13^ Our goal was to develop gene amplification techniques for substantial changes in genome architecture and metabolism. Since multiple mutations usually arise during adaptive evolution, we developed a method for a recipient strain to be transformed by multiple DNA fragments simultaneously on solid medium to confer a selectable phenotype. Together with other methods based on natural transformation, this technique allows the importance of subsets of mutations from complex genetic backgrounds to be revealed rapidly.^26,27^ This work lays the foundation for future biotechnology applications.

## Results and Discussion

### Chromosomal Introduction of a Foreign PCA 2,3-Cleavage Pathway in a PCA^–^/4HB^–^ Host

To alter 4HB and PCA catabolism in *A. baylyi*, foreign *pra* genes from *Paenibacillus* sp. JJ-1b^29^ were introduced into the chromosome. These genes were chosen because they comprise the best studied genetic region encoding a PCA 2,3-cleavage pathway,^29^ they form a contiguous DNA fragment, and they have a similar GC content to the recipient. To prevent use of the native pathway for growth on 4HB or PCA, a recipient strain, an *A. baylyi* mutant (ACN462), was used that cannot synthesize the *ortho* ring-cleaving PCA 3,4-dioxygenase due to a *pcaHG* deletion (Figure 1A).^31^ The foreign DNA was inserted in two chromosomal sites (Figure 2), one of which is within a cluster usually associated with PCA degradation. This ∼70-kbp gene cluster is involved in the degradation of straight chain dicarboxylic acids, aromatic acids, and hydroxylated aromatic acids.^28,32^ This site was chosen because nearly all known genes for aromatic compound degradation by the wild type (ADP1) are in one of three large chromosomal clusters,^32^ and the proximity to other degradative genes could be important. A second insertion site was used that lies outside this cluster, distal to any gene known to be essential (Figure 2B).^33^ After the 4HB^–^ recipient (ACN462) was transformed with linear DNA, drug-resistant transformants were selected in which the *pra* genes were integrated in Site 1 or Site 2 (creating strains ACN1829 and ACN1899, respectively). Both these mutants failed to grow on 4HB or PCA. Details about strains and their construction are in Tables 1 and S1–S3. Some strain lineages are shown in Figure S1, and genetic configurations are in Figure S2.

**Figure 2:**
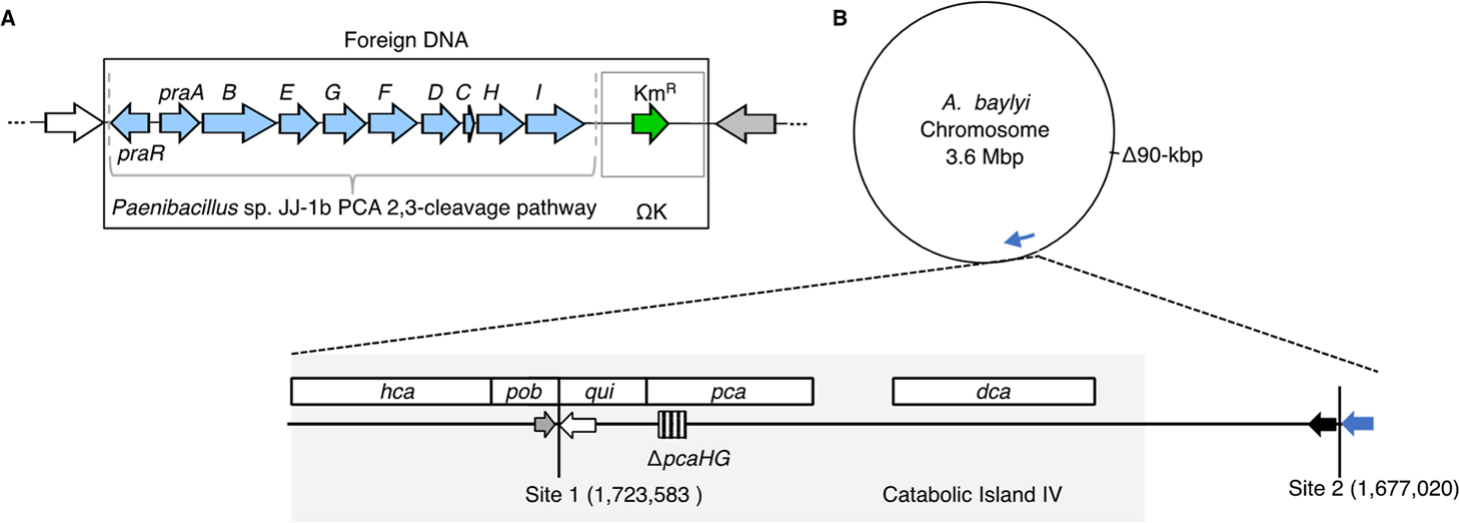
Chromosomal insertions. (A) In an 11.6-kbp region of foreign DNA, the *pra* genes^29^ are adjacent to a kanamycin resistance (Km^R^) cassette (ΩK).^34^ The flanking DNA (white and gray arrows, panel A) matches chromosomal sequences (white and gray arrows, panel B) to enable allelic replacement by homologous recombination. A linear version of this DNA, produced by restriction digestion of a plasmid, was used to transform *A. baylyi* recipients. (B) Schematic chromosomal representation. Two deletions (Δ90-kbp and Δ*pcaHG*) are discussed in the text. An expanded segment (drawn to scale) illustrates the relative positions of two chromosomal integration sites where the *pra* genes were inserted between convergent genes (*quiA* and *pobS*, Site 1) or adjacent genes (ACIAD_RS07700 and ACIAD_RS07705, Site 2). Site 1 is in a gene cluster (gray area), the *hca-pob-qui-pca-dca* supraoperon, involved in aromatic compound catabolism.^22^ Positions and locus tags correspond to the ADP1 genome (NCBI, NC_005966.1). Insertions in both sites are detailed more completely in Figure S2.

**Table 1:** *Acinetobacter baylyi* Strains^a^

strain	relevant characteristics^b^ ^,^ ^c^	source
ADP1	wild type strain (BD413); 4HB^+^	^52^
ACN462	Δ*pcaHG5462*, Δ90-kbp*5462*; 4HB^–^	^31^
ACN1829	Δ*pcaHG5462*, Δ90-kbp*5462*, *praRABEGFDCHI*-ΩK*51829*; 4HB^–^	this study
ACN1842	Δ*pcaHG5462*, Δ90-kbp*5462*, *praRABEGFDCHI*-ΩK*51829* (in Site 1), amplified population (has SBF*51959* junction); 4HB^+^	this study
ACN1899	Δ*pcaHG5462*, Δ90-kbp*5462*, ΩK-*praRABEGFDCHI51899*; 4HB^–^	this study
ACN1936	Δ*pcaHG5462*, Δ90-kbp*5462*, *praRABEGFD(C)HI*-ΩK*51936*, *praC51936*, Δ*pobS*-*hcaC51936*, *rpoB51936*, *pbpA51936*, *ACIAD_RS01270* *51936*, *ACIAD_RS06205 51936* (Table S4, isolate arose during evolution of the ACN1842 population); 4HB^+^	this study
ACN1939	Δ*pcaHG5462*, Δ90-kbp*5462*, *praRABEGFD(C)HI-*ΩK*51936*, *praC51936*, additional mutations; 4HB^+^	this study
ACN1956	Δ*pcaHG5462*, Δ90-kbp*5462*, ΩK*-praRABEGFDCHI51899*, amplified population (has SBF*51961* junction); 4HB^+^	this study
ACN1992	Δ*pcaHG5462,* Δ90-kbp*5462*, *praRABEGFD(C)HI*-ΩK*51936*, *praC51936*, *pobR51992*, additional mutations; 4HB^+^	this study
ACN1993	Δ*pcaHG5462,* Δ90-kbp*5462*, *praRABEGFD(C)HI*-ΩK*51936*, *praC51936*, additional mutations; 4HB^+^	this study
ACN2045	Δ*pcaHG5462*, Δ90-kbp*5462*, *praRABEGFDCHI*-ΩK*51829*, amplified population (has SBF*51959* junction); 4HB^+^	this study
ACN2235	*pcaK52106*, Δ*pcaUIJFBDCHG52106*, *praRABEGFD(C)HI*-ΩK*52611*, *praC52611*, *pobA*::ΩS*52212*; 4HB^–^	this study
ACN2237	*pcaK52106*, *ΔpcaUIJFBDCHG52106*, *praRABEGFD(C)HI*-ΩK*52237*, Δ*praC52237*, *pobA*::ΩS*52212*; 4HB^–^	this study
ACN2271	*pcaK52106*, Δ*pcaUIJFBDCHG52106*, *pobA*::ΩS*52212*, *praRABEGFD(C)HI*-ΩK*52611*, *praC52611*, *gacS52271*; 4HB^+^	this study
ACN2276	*pcaK52106*, Δ*pcaUIJFBDCHG52106*, *pobA*::ΩS*52212*, *praRABEGFD(C)HI*::ΩK*52237*, Δ*praC52237*, *gacA52276*; 4HB^+^	this study
ACN2504	P_d5h6_-*pcaK52401*, Δ*pcaUIFJBDKCHG52401*, *praRABEGFDCHI*-ΩK*51829*; 4HB^–^	this study
ACN2506	P_D5H6_-*pcaK52401*, Δ*pcaUIFJBDKCHG52401*, Δ*pobA52440*, *praRAB(C)EGFDH(I)*-ΩK*52506*, Δ*praC52237*, Δ*praI52506*, *gacA52276*, *sacB*-ΩS*52474*; 4HB^–^	this study
ACN2557	P_D5H6_-*pcaK52401*, Δ*pcaUIFJBDKCHG52401*, Δ*pobA52440*, *praRABEGFD(C)HI-ΩK52237*, Δ*praC52237*, *gacA52276*; 4HB^+^ on plates, 4HB^–^ in liquid	this study
ACN2608	Δ*pcaHG5462*, Δ90-kbp*5462*, ΩK-*praRABEGFD(C)HI52608*, *praC52608* Table S5), additional uncharacterized mutations; 4HB^+^ Isolate from evolving population of ACN1956	this study
ACN2611	Δ*pcaHG5462*, Δ90-kbp*5462*, *praRABEGFD(C)HI*-ΩK*52611*, *praC52611* (Table S5), additional uncharacterized mutations; 4HB^+^ Isolate from evolving population of ACN2045	this study
ACN3315	Δ*pcaHG5462*, Δ90-kbp*5462*, *praRAB(C)EGFDCHI*-ΩK*53315*, *praC53315*, Δ*gacA53315*, Δ*pobA52440*, *relA53315*; 4HB^+^	this study
ACN3316	Δ*pcaHG5462*, Δ90-kbp*5462*, *praRABEGFD(C)HI*-ΩK*52237*, Δ*praC52237*, Δ*gacA53315*, Δ*pobA52440*; 4HB^+^	this study
ACN3317	Δ*pcaHG5462*, Δ90-kbp*5462*, *praRABEGFD(C)HI-*ΩK*52237*, Δ*praC52237*, Δ*gacA53315*; 4HB^+^	this study
ACN3318	Δ*pcaHG5462*, Δ90-kbp*5462*, *praRAB(C)EGFDCHI-*ΩK*52237* , Δ*praC52237*, *gacS53318*, Δ*pobA52440*, *benK53318*; 4HB^+^	this study
ACN3326	P_D5H6_-*pcaK52401*, Δ*pcaUIFJBDKCHG52401*, Δ*pobA52440*, *praRABEGFD(C)HI*-ΩK*52237*, Δ*praC52237*, *gacA52276*, Δ90-kbp*5462*; 4HB^+^	this study
ACN3419	P_D5H6_-*pcaK52401*, Δ*pcaUIFJBDKCHG52401*, Δ*pobA52440*, *praRAB(C)EGFDH(I)*-ΩK*52506*, Δ*praC52237*, Δ*praI52506*, Δ*gacA53315*, Δ31-kbp*53419*; 4HB^–^	this study
ACN3444	Δ*pcaHG5462*, Δ90-kbp*5462*, *praRABEGFD(C)HI*-ΩK*52237*, Δ*praC52237*, Δ*gacA53315*, Δ*pobA52440*, Δ*csrA*::ΩS*53444*; 4HB^–^	this study
ACN3496	Δ*pcaHG5462*, Δ90-kbp*5462*, *praRABEGF(DC)HI-*ΩK*53496*, Δ*praDC53496,* Δ*gacA53315*, Δ*pobA52440*; 4HB^–^	this study
ACN3515	Δ*pcaHG5462*, *praRABEGFDCHI*-ΩK*51829*	this study

a *A. baylyi* strains were derived from ADP1, previously known as *Acinetobacter* sp. or *Acinetobacter calcoaceticus*. Additional strain construction details are provided in Tables S1–S3.

b Numbers in bold correspond to chromosomal positions in the wild-type ADP1 sequence (NCBI entry NC_005966).

c Genes in parentheses carry mutations or deletions. Drug-resistance omega cassettes are indicated by ΩK and ΩS.^34,55^ Two large deletions discussed in the text are indicated by Δ90 kbp*5462*, a 90,164-bp spontaneous chromosomal deletion (944,926-1,035,015), and Δ31 kbp*53419*, a 30,830-bp engineered chromosomal deletion (944,728–975,557).

Genome sequencing revealed that the new strains, and the Δ*pcaHG* parent from which they were derived, all had a 90,164-bp deletion (hereafter called Δ90-kbp, Figure 2B) in a region known to undergo spontaneous deletion.^35^ To test whether this deletion prevents the *pra* genes from functioning, a strain (ACN3515) was made with wild-type DNA in the 90-kbp region that was otherwise isogenic to that with *pra* genes in Site 1 (ACN1829). To ensure 4HB uptake, another strain was made, also with wild-type DNA in the 90-kbp region and the *pra* genes in Site 1. In this strain (ACN2504), all genes for the native PCA 3,4-cleavage pathway were replaced with a constitutively transcribed 4HB transporter gene, *pcaK*.^9,36^ All these strains were 4HB^–^, and no 4HB^+^ derivatives arose by direct selection. Thus, one chromosomal copy of the *pra* genes failed to support growth on 4HB regardless of Δ90-kbp or constitutive PcaK expression.

### Multiple Copies of the Nonnative Pathway Enabled Growth on 4HB

Since the lack of growth might result from insufficient enzyme production, we used the EASy method to enable variable *pra*-gene dosage.^10–12^ Strains were transformed with linear DNA, called the synthetic bridging fragment (SBF), to promote duplication of the *pra*-gene segment by homologous recombination (Figure 3).

**Figure 3:**
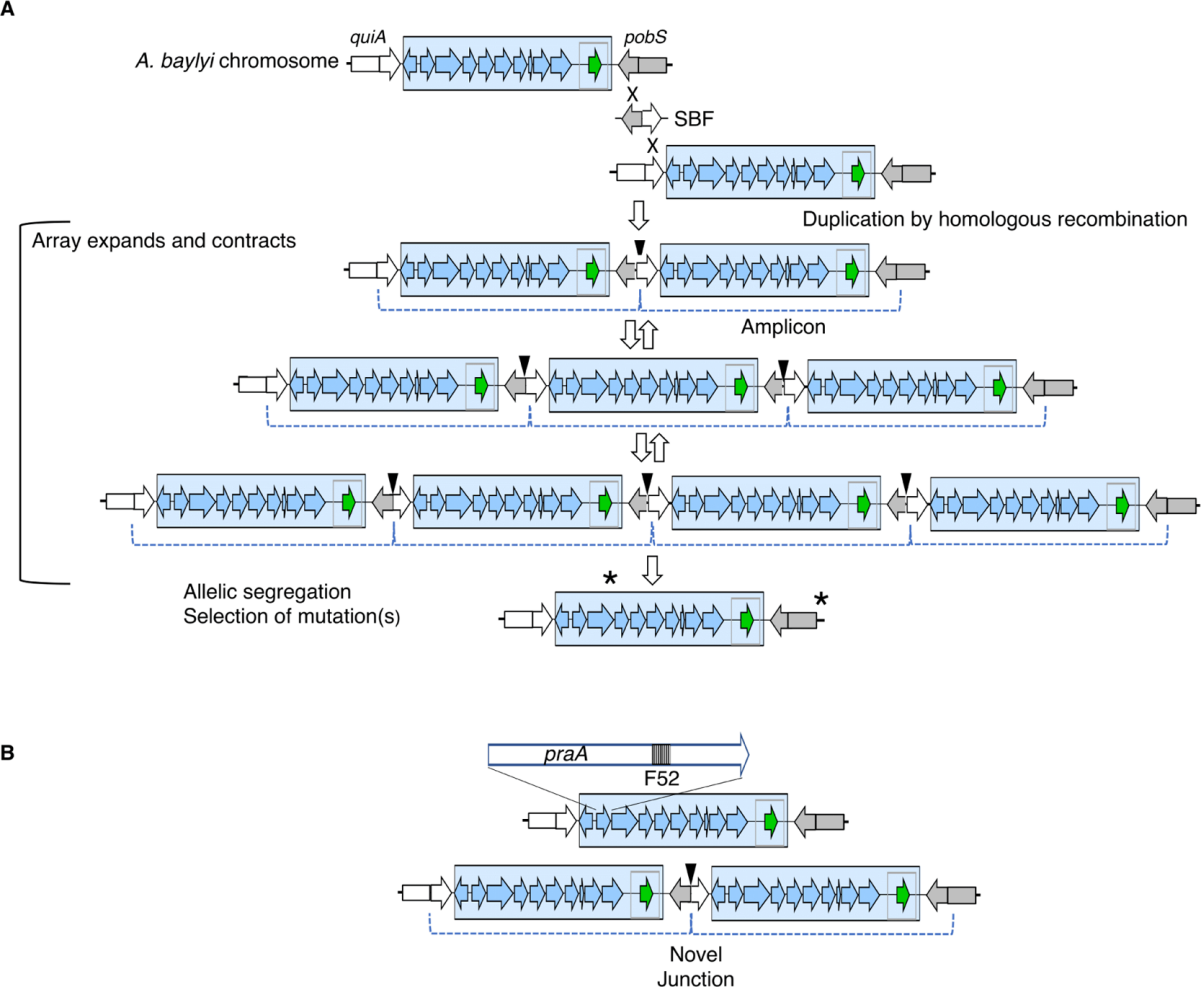
EASy amplification of foreign DNA in chromosomal Site 1. (A) Recipients were transformed by a linear synthetic bridging fragment (SBF) that can undergo homologous recombination (X) to duplicate a chromosomal fragment (the amplicon, dotted bracket). Amplicon copy number can vary via recombination and is determined by selective advantage. Transformants were first selected using a drug concentration that requires more than one copy of the Km^R^ cassette (green arrow). Next, populations were selected for growth on 4HB without Km. During growth on 4HB, as the array could expand or contract, beneficial mutations arose within the amplified region and elsewhere on the chromosome (not depicted). Over time, mutations (asterisks) were selected that obviated the benefit of multiple copies, and allelic segregation occurred. (B) PCR primers specific to *praA* were used in qPCR to detect a 52-bp fragment (F52) and quantify its abundance relative to a DNA fragment outside the amplicon that is presumed to be in single copy. The novel junction in the chromosome created by the SBF (black triangles) can be detected by PCR. This junction is only in the genome of strains that have duplication/amplification of the target region. This figure illustrates Site 1, and the same techniques were used for Site 2 (using a different SBF and generating a different novel junction, Figure S2).

SBF transformants, derived from strains with the *pra* genes, were selected with Km at a concentration requiring multiple copies of the resistance gene. Selection for high-level drug resistance yielded only mutants with increased dosage of the target region. Next, cells were transferred to Km-free 4HB plates, and 4HB^+^ colonies were isolated. Thus, the PCA 2,3-cleavage pathway could become functional after augmentation of Pra enzyme expression resulting from amplification of the *pra* genes. Three independent 4HB^+^ populations were obtained (ACN1842, ACN1956, and ACN2045). These populations were serially transferred in liquid culture to select faster growth on 4HB during adaptive laboratory evolution. A sequence in the *pra*-gene cassette was used as a proxy for amplicon copy number (F52, Figure 3), and copy number initially ranged from 13 to 76 (Figure 4). After 7–20 weeks of serial transfer (approximately 150 to 450 generations), each population had an average of one F52 copy, suggesting mutations had occurred that alleviated the need for multiple copies of the *pra* genes.

**Figure 4:**
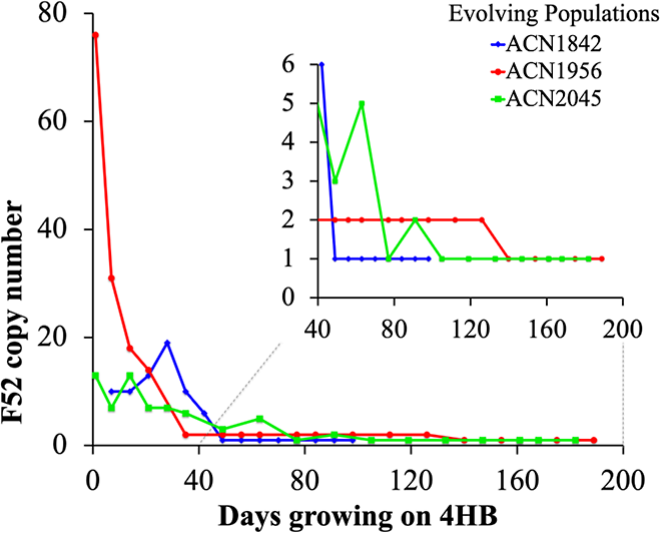
Average copy number of a fragment in *praA* (F52) assessed by qPCR. Samples of three evolving populations were analyzed at different times during serial transfer in medium with 4HB as the carbon source. Each population (ACN1842, ACN1956, and ACN2045) was found to have a single copy of F52 at day 49, 140, or 77, respectively. The *pra* genes in ACN1842, ACN1956, and ACN2045 are in Site 1, Site 2, and Site 1, respectively. Information about qPCR efficiency is provided in Figure S3. The standard deviations of replicates of each qPCR reaction differed by no more than 20% of the value.

### Mutations in 4HB^+^ Isolates that Grow Using the Nonnative PCA 2,3-Cleavage Pathway

One 4HB^+^ colony from each population was isolated after the copy number declined. Each strain was named and confirmed to have a single F52 copy. Genome sequencing revealed that all three strains (ACN1936, ACN2608, and ACN2611) had multiple mutations that distinguished them from their 4HB^–^ parents. The mutations of two of these strains, ACN2611 and ACN2608, included rearrangements of the amplicon that resulted in one copy of the *praR-praA* region but multiple copies of some other *pra* genes, complicating the analyses. The mutations of ACN1936 (derived from ACN1829) are shown in Table S4.

We sought to identify mutation(s) sufficient for growth on 4HB with a single copy of *pra* genes. Only one gene was mutated in all strains, *praC*. Three different alleles were identified, each causing a reading-frame shift affecting the normally small (63 amino acid) PraC. The encoded variants were altered after amino acid residue 24, 52, or 58 (Table S5). These sequence changes suggested that the variants would be dysfunctional. However, *praC* mutations were surprising since the encoded tautomerase is predicted to be required for growth (Figure 1).

The *pra* region with a mutated *praC* (allele *praC51936*) and its adjacent drug-resistance cassette was recovered from a 4HB^+^ strain (ACN1936), using the gap-repair method.^26,37^ This DNA was introduced into the chromosome of a 4HB^–^ parent (ACN462) by allelic replacement. With heavy inoculation of the new strain on medium with 4HB as the carbon source, some colonies arose after a delay. The small number of colonies and the delay in their appearance suggested that *praC* mutation alone is insufficient for growth. After streak purifying three such 4HB^+^ strains (ACN1939, ACN1992, ACN1993), genome sequencing confirmed that each carried *praC51936* but also additional strain-specific mutations. Since comparable experiments with the wild-type *praC* never led to 4HB^+^ derivatives, *praC* mutation likely confers a benefit for growth on 4HB.

In the three spontaneous 4HB^+^ strains with *praC51936*, the only consistently mutated region encompassed divergently transcribed *pobR* and *pobA*, genes located in catabolic island IV (Figure 2B). PobA, a native hydroxylase that converts 4HB to PCA, is functionally redundant to PraI (Figure 1). The transcriptional regulators of these genes, PobR and PraR, respectively, also have corresponding functions. Each strain had a different mutation in *pobR* or *pobA*, in a region that was entirely deleted in the strain where this *praC* allele arose (ACN1936).

To determine whether mutations in both *praC* and *pobA* confer a 4HB^+^ phenotype, two new strains were constructed. These strains carry a *pobA* disruption (allele *pobA*::ΩS*52212*) and additionally a *praC* mutation. In both new strains, the *pra*-genes were in Site 1 of the chromosome, and native *pca* genes were deleted. In one of the new strains (ACN2237) a *praC* deletion (allele Δ*praC52237*) was introduced. In the other strain (ACN2235), we introduced a previously identified *praC* mutation (allele *praC52611*). Neither combination of mutations in *pobA* and *praC* enabled the engineered strains to grow on 4HB. However, direct inoculation of many cells from these new strains (ACN2235 and ACN2237) on solid medium yielded 4HB^+^ colonies, presumably with additional spontaneous mutations. Genome sequencing of two such 4HB^+^ isolates revealed that each had a mutation in *gacA* or *gacS*, genes that encode partners in a two-component transcriptional regulatory system.^38^ A nonsense mutation causing early truncation of the response regulator, GacA, was identified in one 4HB^+^ strain (ACN2276, derived from ACN2237). A frameshift in *gacS*, which encodes the histidine kinase of the regulatory system, was identified in the other 4HB^+^ strain (ACN2271, derived from ACN2235).

To test the effect of a defined combination of mutations, deletions of *praC*, *pobA*, and *gacA* were introduced into a 4HB^–^ parent, ACN1829 (Δ*pcaHG*, Δ90-kbp, foreign DNA in Site 1). These three engineered changes caused the resulting strain (ACN3316) to become 4HB^+^ without acquiring any additional mutations. Thus, the deletion of three genes (*praC*, *pobA*, and *gacA*) proved sufficient to enable functionality of the nonnative PCA 2,3-cleavage pathway encoded by one copy of the *pra*-gene set (without *praC*) in Site 1 of the *A. baylyi* chromosome.

### Transformation Assay to Assess Combinations of Mutations Sufficient for a 4HB^+^ Phenotype

The identification of a combination of mutations that enable growth on 4HB required several iterations of reverse engineering, screening, selection, and genome sequencing. While the unique genetic system of *A. baylyi* facilitated the process, such intensive effort is not always practical or sufficient. Therefore, we sought to develop a transformation assay that would simplify mutational analyses. A method was developed 40 years ago for natural transformation and selection by dropping cell-free DNA fragments directly on the surface of a selective-medium plate spread with recipient cells.^39^ Colonies grow in spots where natural transformation by the donor DNA introduces appropriate chromosomal changes. This method of lawn transformation has been used to evaluate individual mutations.^26^ Since no individual mutation enabled 4HB consumption via the PCA 2,3-cleavage pathway, we tested mixtures of donor DNA. This method, termed CEMENT (Combinatorial Evaluation of Mutations Examined by Natural Transformation), was developed with different combinations of alleles identified in 4HB^+^ mutants: *praC*, *gacA* (or *gacS*), and *pobA* (or *pobR*). Two recipients were tested that each had one copy of the *pra* genes in Site 1 of the chromosome. These recipients (ACN1829 and ACN2504) varied in the specific *pca*-gene deletion interrupting the native pathway, the promoter controlling *pcaK*, and the presence or absence of the Δ90-kbp.

In one example (Figure 5), 4HB^–^ recipient cells (ACN2504) were spread on the surface of 4HB solid medium. Cell-free DNA fragments with mutations in *gacA*, *pobA*, and/or *praC* were dropped in marked regions on top of the cells. Each linear fragment had flanking DNA (of approximately 1 kbp or more) matching the chromosome to enable allelic replacement. Growth occurred only in region 7, where three donor DNA fragments were dropped. This mutational combination appears to be sufficient for the foreign pathway to function. Genomic DNA sequence of a streak-purified 4HB^+^ transformant confirmed that it acquired chromosomal sequences identical to the three mutated donor DNA fragments and no additional mutations.

**Figure 5:**
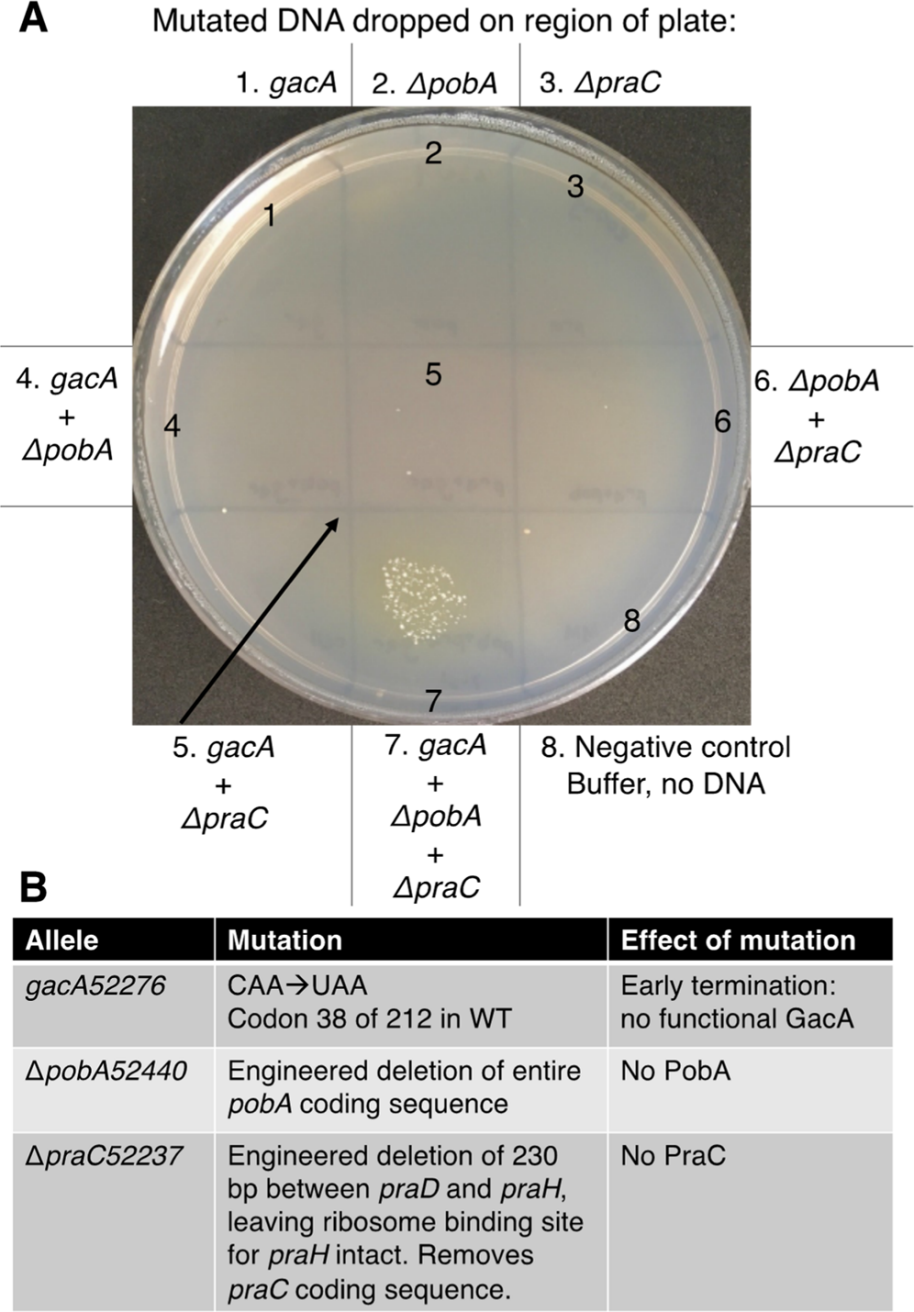
Transformation assay (CEMENT) to assess which combinations of mutations in three genes confer growth on 4HB using the PCA 2,3-cleavage pathway. (A) A sample of a 4HB^–^ recipient culture (ACN2504) was spread on the surface of a Petri dish with 4HB as the carbon source. In the center of marked regions of the plate, linear (cell-free) DNA was dropped on top of the recipient cells. This DNA carries mutations that eliminate the function of *gacA*, *pobA*, and *praC* (allele designations *gacA52276*, Δ*pobA52440*, and Δ*praC52237*). The mutation on the fragment is surrounded by flanking DNA that matches the chromosome to allow allelic replacement by homologous recombination. (B) The mutations either arose during adaptive evolution (*gacA52276*) or were engineered to delete genes in which mutations accrued during adaptive evolution (Δ*pobA52440* and Δ*praC52237*). Linear donor DNA was generated by digesting the following plasmids using restriction enzymes: pBAC1711 (*gacA5227*), pBAC1578 (Δ*pobA52440*), and pBAC1642 (Δ*praC52237*).

In different experiments, results were comparable whether the donor DNA carried an allele isolated from a 4HB^+^ mutant or an engineered deletion of the same gene. For example, DNA with the *praC* point mutation from ACN1936 (*praC51936*) was comparable to DNA with a *praC* deletion (Δ*praC52237*). Thus, all the spontaneous mutations appear to cause loss-of-function for the loci investigated. Similarly, since the effects were comparable for *pobA* and *pobR* mutations or deletions, all selected mutation(s) appear to reduce or eliminate PobA expression. Furthermore, the results with mutations that prevented GacA expression were comparable to those with mutations that prevented GacS expression, consistent with both components of the two-component transcriptional regulatory system affecting the phenotype.

When the recipient had Δ90-kbp (ACN1829), 4HB^+^ colonies occasionally arose in spots where two DNA fragments were added as well as in the spot where all three were added. Several 4HB^+^ colonies were streak purified, each corresponding to a transformant arising from every combination of two or three donor fragments in CEMENT assays (with ACN1829 as recipient). In these experiments, all donor fragments were engineered deletions of *pobA*, *praC*, and/or *gacA*. Whole genome sequencing of several transformants confirmed that each acquired the alleles of the donor DNA (Table S6). A strain (ACN3316) with deletions of *pobA*, *praC*, and *gacA* had no other mutations relative to the recipient strain.

In two strains that acquired two rather than three of the donor DNA mutations, the resulting 4HB^+^ transformants had a spontaneous mutation in the third gene. For example, the strain transformed by Δ*gacA* and Δ*pobA* had a 1-bp insertion in *praC* (ACN3315). Similarly, a spontaneous 1-bp deletion in *gacS* was selected in the strain transformed with Δ*pobA* and Δ*praC* (ACN3318). In contrast, a strain that acquired only Δ*gacA* and Δ*praC* grew on 4HB with no additional mutations (ACN3317). Subsequent experiments indicated that when mutations in both *gacA* and *praC* were present, there was a requirement for an additional *pobA* (or *pobR*) mutation if, and only if, wild-type DNA was present in the 90-kbp region. As described later, the significance of Δ90-kbp was explored further. Collectively these results highlight the importance of mutations that eliminate the function of PraC, the two component GacA-GacS system, and, in some cases, PobA (or PobR).

While these mutations were sufficient for the consumption of 4HB as the sole carbon source via the PCA 2,3-cleavage pathway, growth was typically 2- to 5-fold slower than that for the wild type using its native pathway (Figure S4). Applications for strains engineered to use this nonnative pathway are expected to involve the metabolism of aromatic compound mixtures rather than 4HB as a sole carbon source. Further metabolic optimization of strains may be required to achieve targeted outcomes. Next, our attention was directed toward understanding the basis of the phenotypic change conferred by a small set of mutations.

### Key Mutations: Loss of a Tautomerase (PraC) Facilitates 4HB Consumption

Every characterized 4HB^+^ strain carried a *praC* mutation. Since the encoded tautomerase is predicted to catalyze an integral reaction in the PCA 2,3-cleavage pathway, *praC* mutations were unexpected. We considered the possibility that this enzymatic step could be bypassed in a manner that occurs in a pathway encoded by a plasmid from *Pseudomonas putida* mt-2 (pWW0).^40,41^ In this alternate pathway, three enzymes comparable to PraB, PraC, and PraD are replaced by a single hydrolase, encoded by *xylF* (dashed arrow in Figure 1). If such a bypass occurred in the *A. baylyi* mutants, then PraB and PraD, like PraC, would be unnecessary for growth on 4HB. To test this possibility, a strain was constructed with a *praD* deletion. This strain (ACN3496) was otherwise isogenic to a 4HB^+^ strain (ACN3316, Table S6). However, the deletion prevented growth on 4HB, suggesting a requirement for PraD that would rule out a hydrolase-mediated bypass. A different possibility is that PraC causes one or more metabolite(s) to accumulate to toxic levels during heterologous expression. Decreasing the rate of the reaction usually mediated by PraC, perhaps through nonenzymatic tautomerization, may reduce bottlenecks during 4HB degradation. While tautomerization could be mediated by a native *A. baylyi* enzyme, homology searches failed to identify a PraC-like candidate.

### Key Mutations: Loss of the GacA/GacS Two-Component System Facilitates 4HB Consumption

Loss of GacA or GacS contributed to the 4HB^+^ phenotype of strains using the PCA 2,3-cleavage pathway. This two-component system, known by several different designations in diverse bacteria, controls varied functions in *Acinetobacter baumannii*, a pathogenic species.^38,42,43^ While the role of this system has not been reported in *A. baylyi*, well-conserved roles of GacA/GacS homologues raise questions about the possible connection to a small RNA-binding protein, CsrA.^44^ The GacA/GacS system often activates transcription of noncoding RNAs capable of sequestering CsrA, a post-transcriptional regulator. Moreover, mutations in *csrA* have been found to affect fitness in evolutionary studies of *A. baylyi*.^45^ We investigated whether the 4HB^+^ phenotype conferred by *gacA* or *gacS* deletion depends on CsrA.

Our model proposes that loss of GacA/GacS would eliminate or reduce the amount of noncoding RNAs that bind and sequester CsrA. In turn, more CsrA would be available to regulate target transcript(s). If increased CsrA availability is a critical aspect of the *gacA*/*gacS* mutations, the 4HB^+^ phenotype would be expected to depend on CsrA. We tested this prediction by replacing *csrA* with a drug-resistance marker in a 4HB^+^ parent strain (ACN3316) that grows via the PCA 2,3-cleavage pathway (and carries alleles Δ*gacA53315*, Δ*pobA52440*, and Δ*praC52449*). When *csrA* was deleted from this parent, the resulting strain (ACN3444) lost the ability to grow on 4HB. In contrast, both strains grew on pyruvate. Therefore, the 4HB^+^ phenotype conferred (in part) by the loss of GacA/GacS appears to require CsrA. This result is consistent with the hypothesis that growth via the foreign pathway is facilitated by CsrA-regulated target(s) that have yet to be identified.

### Key Mutations: The Benefit of Inactivating PobA Depends on Genetic Context

Mutations in the *pobA-pobR* region were selected during growth on 4HB in at least four distinct lineages. Mutations in the *pobR* regulatory gene appeared to reduce or eliminate *pobA* expression, as indicated by the identical effects of deleting *pobR* or *pobA* in the CEMENT assays. PobA, a 4HB hydroxylase that converts 4HB to PCA, catalyzes the same reaction as PraI (Figure 1). For growth on 4HB, at least one hydroxylase is required to produce PCA. However, it is not evident why retaining both enzymes during growth on 4HB should be problematic.

Despite their functional similarities, the hydroxylase homologues have different cofactor specificities.^46,47^ PobA depends on NADPH, while PraI can use NADH or NADPH. All selected spontaneous mutations inactivated *pobA* and not *praI*. Since the rate of PCA production depends on this enzyme, the possibility was raised that this difference explains the preference for PraI during degradation of 4HB via the PCA 2,3-cleavage pathway, as observed in engineered strains of *P. putida* KT2440.^47^ Another factor that might contribute to preferential use of PraI is genetic location. The *praI* gene, located at the end of the *pra* operon (Figure 2), is cotranscribed with other pathway genes. In contrast, *pobA* is in the vicinity of the *pra* genes but is not transcribed with them.

A transformation assay was used to investigate the importance of genetic location. The recipient strain had deletions of both *praI* and *pobA* (Figure 6A). As expected, this strain (ACN2506) is 4HB^–^. Donor DNA was engineered to insert *praI* in the chromosome by allelic replacement into either the original *praI* locus or the *pobA* locus. The integration site was determined by the flanking sequences that direct homologous recombination between donor and chromosomal DNA. Similarly, fragments were engineered to insert *pobA* into either locus. Linear donor DNA was dropped in spots on top of the recipient strain, which was spread across solid 4HB medium.

**Figure 6:**
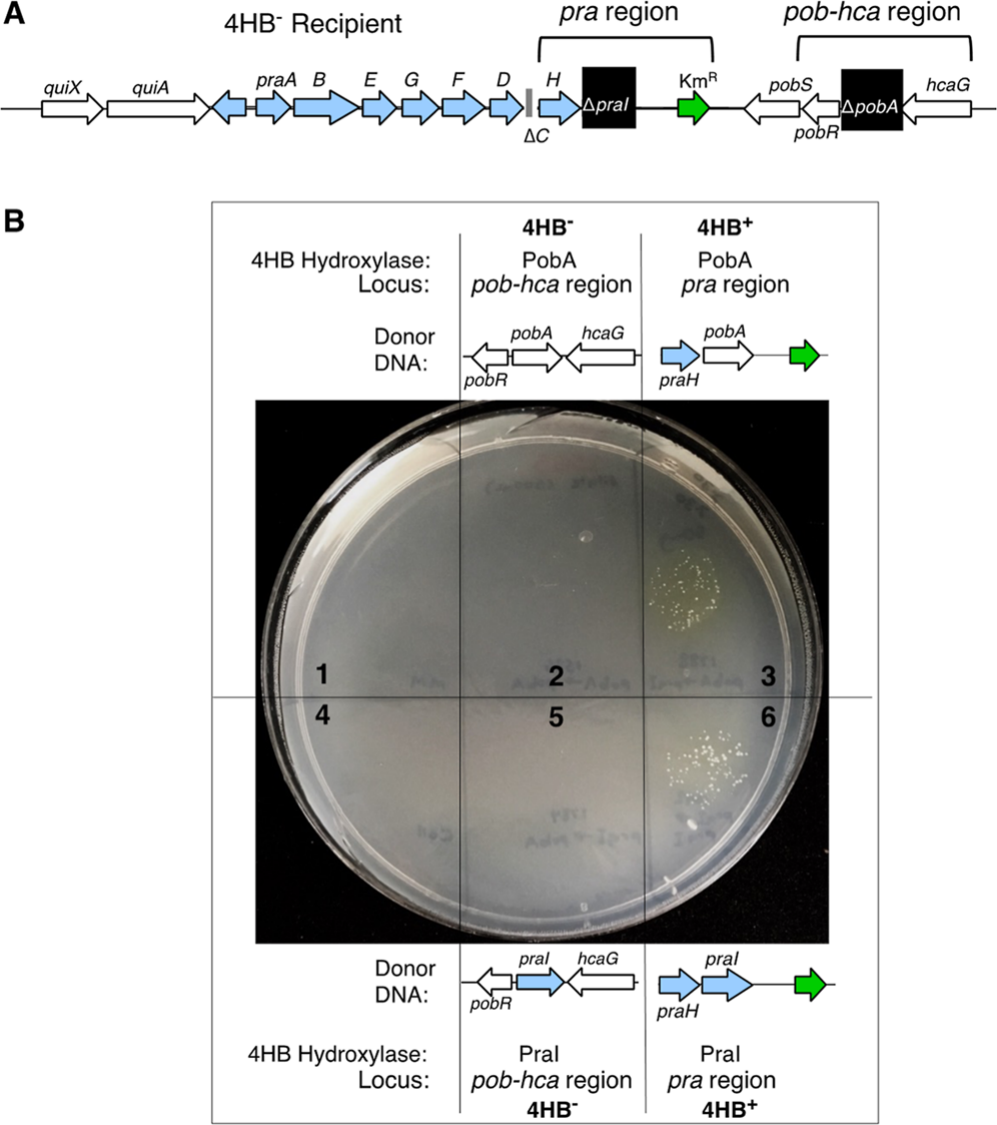
Integration of hydroxylase genes in two loci. (A) Chromosomal region of the *praI* and *pobA* deletions that cause the recipient (ACN2506) to be 4HB^–^. A 4HB^+^ phenotype should result from expression of a functional 4HB hydroxylase because of the additional mutations in this strain (Δ*praC* and disrupted *gacA*) (B) After this recipient (ACN2506) was spread on a 4HB plate, linear donor DNA fragments were dropped in spots. Allelic replacement should integrate *pobA* in its native locus (spot 2), *pobA* in place of *praI* (spot 3), *praI* in place of *pobA* (spot 5), and *praI* in place of its deletion (spot 6). Spots 1 and 4 were negative controls. Colonies arise where transformation by the donor DNA confers a 4HB^+^ phenotype.

In spots where the donor DNA could replace the *praI* deletion with either *praI* or *pobA*, 4HB^+^ transformants arose (Figure 6B, spots 3 and 6). In contrast, in spots where the donor DNA could replace the *pobA* deletion with either *praI* or *pobA*, no 4HB^+^ transformants arose (Figure 6B, spots 2 and 5). Thus, either *pobA* or *praI* can confer growth on 4HB when positioned at the end of the *pra* operon, which indicates that either enzyme can enable growth via the PCA 2,3-cleavage pathway. There is no strict requirement for the PraI-type of 4HB hydroxylase. The results suggest that coexpression of the hydroxylase with other Pra enzymes is important. Similarly, the failure of both genes to confer 4HB^+^ growth when each was targeted to the *pobA* locus suggests insufficient expression from this locus. These results do not reveal the basis of the selective pressure to inactivate *pobA* during 4HB^+^ growth. However, the importance of genetic context may reflect regulatory interference. Improperly regulated hydroxylase expression could result in too much (or too little) production of PCA, which in turn could cause the toxic accumulation (or insufficient production) of this or another metabolite. While additional studies are needed to confirm the underlying problem, the results described below suggest that mutations in *pobA* are selected in response to conditions involving high-level 4HB uptake, implicating a high rate of PCA production as being deleterious.

### Key Mutations: a 90-kbp Chromosomal Deletion Affects Growth on 4HB

In the CEMENT experiments, only strains with the Δ90-kbp grew on 4HB without mutations in *pobA* or *pobR*. While exploring this observation, an interesting disparity was discovered between growth on 4HB as the carbon source in liquid medium compared to solid medium. This disparity, which occurred only in strains with wild-type DNA in the 90-kbp chromosomal region, yielded 4HB^+^ colonies on agar plates that were 4HB^–^ in liquid culture. We studied this unusual result by trying to identify genetic changes that would enable growth in both types of 4HB media. As the recipient in a transformation assay, we used a strain that grew on 4HB on solid medium but not in liquid. In this recipient (ACN2557), mutations in *gacA*, *praC*, and *pobA* contribute to the 4HB^+^ phenotype of colonies. This strain was transformed on a plate using cell-free genomic DNA from a strain (ACN1829) that has the Δ90-kbp but fails to grow on 4HB under all growth conditions. This donor DNA harbors wild-type alleles of *gacA*, *praC*, and *pobA*. After transformation, colonies from solid 4HB medium were used to inoculate liquid 4HB medium. After selection for growth in liquid, a transformant (ACN3326) was isolated that was 4HB^+^ on both types of media. Whole genome sequencing revealed that this transformant differed from the recipient (ACN2557) only in that it had acquired the Δ90-kbp of the donor DNA. Thus, wild-type DNA in the 90-kbp region of the recipient appears to prevent growth on 4HB in liquid, whereas deletion of this DNA enabled the transformant to grow.

To narrow the region responsible for the 4HB growth disparity, constructs were made to delete specific ∼30-kbp segments that collectively span the 90-kbp region of interest (Figure S5). Each engineered deletion was used to transform a recipient that is 4HB^+^ on agar but 4HB^–^ in liquid (ACN2557). Cells were transformed on 4HB plates in experiments with each donor fragment that encompassed one of the three ∼30-kbp deletions. Transformants were transferred to liquid 4HB medium. Only one of these regions generated transformants that grew on both types of 4HB media. Thus, the DNA responsible for the 4HB^+^ or 4HB^–^ phenotype in liquid was narrowed to a 31-kbp DNA segment predicted to encode 30 proteins, listed in Table 2.

**Table 2:** Chromosomal Region^a^ Affecting Ability of Some Strains to Grow in 4HB Liquid Medium^b^

locus tag (genome position)	gene name	predicted gene product
ACIAD_RS04410 (943,626 ← 944,785)		IS*1236* family transposase
ACIAD_RS04420 (944,858 ← 945,619)		YrzE family protein (pseudogene)
ACIAD_RS04425 (946,208 → 947,596)		succinate-semialdehyde dehydrogenase
ACIAD_RS04430 (947,697 ← 948,011)		hypothetical protein
ACIAD_RS04435 (948,144 ← 948,884)		NUDIX hydrolase
ACIAD_RS04440 (949,007 ← 950,524)		nicotinate phosphoribosyltransferase
ACIAD_RS04445 (950,541 ← 951,422)		ribose-phosphate pyrophosphokinase
ACIAD_RS04450 (951,754 → 953,685)		phosphatase PAP2 family protein
ACIAD_RS04455 (953,779 ← 954,372)		amino acid transporter
ACIAD_RS04460 (954,585 →955,478)		ArgP/LysG family transcriptional regulator
ACIAD_RS04465 (955,578 → 956,267)		GntR family transcriptional regulator
ACIAD_RS04470 (956,269 ← 957,033)		ABC transporter ATP-binding protein
ACIAD_RS04475 (957,037 ← 958,053)		iron ABC transporter permease
ACIAD_RS04480 (958,057 ← 959,082)		ABC transporter substrate-binding protein
ACIAD_RS04485 (959,235 → 961,349)		TonB-dependent receptor
ACIAD_RS04490 (961,392 ← 961,847)		GNAT family N-acetyltransferase
ACIAD_RS04495 (962,614 → 963,069)		hypothetical protein
ACIAD_RS04500 (963,641 → 964,639)		isopenicillin N synthase family oxygenase
ACIAD_RS04505 (964,806 → 965,492)	*vanR*	GntR family transcriptional regulator
ACIAD_RS04510 (965,539 ← 966,486)	*vanB*	PDR/VanB family oxidoreductase
ACIAD_RS04515 (966,498 ← 967,574)	*vanA*	aromatic ring-hydroxylating dioxygenase
ACIAD_RS04520 (968,383 → 969,729)	*vanK*	MFS transporter
ACIAD_RS04525 (969,761 → 971,044)	*vanP*	OprD family outer membrane porin
ACIAD_RS04530 (971,137 ← 972,276)		FAD-dependent monooxygenase
ACIAD_RS04535 (972,542 ← 973,354)		IclR family transcriptional regulator
ACIAD_RS04540 (973,629 → 973,934)		nonheme iron oxygenase ferredoxin subunit
ACIAD_RS04545 (973,953 → 974,252)		hypothetical protein
ACIAD_RS04550 (974,270 → 975,322)		aromatic ring-hydroxylating dioxygenase alpha subunit

a This region corresponds to that deleted in Δ31 kbp53419, 944,728 – 975,557.

b Locus tags, genome positions, and predicted functions correspond to those features on the ADP1 chromosome in NCBI entry NC_005966.

We tested the effect of this deletion (designated Δ31-kbp) on the other known impact of Δ90-kbp. Namely, deletion of the 90-kbp region can alleviate the requirement for *pobA*/*pobR* mutations in 4HB^+^ strains growing via the PCA 2,3-cleavage pathway (when there are *praC* and *gacA*/*gacS* mutations). A transformation assay, comparable to that in Figure 6, was conducted with a Δ31-kbp recipient (ACN3419). In contrast to what was observed with a recipient carrying wild-type DNA in this region (Figure 6), results with the Δ31-kbp recipient (ACN3419) demonstrated functionality of *pobA* whether it was integrated in place of *pobA* or in place of *praI* (Figure S6). In addition, *praI* enabled growth when expressed from the *pobA* locus. These results suggest that DNA in the region of Δ31-kbp can impact the regulated hydroxylation of 4HB.

Within this 31-kbp region are several *van* genes encoding proteins for the consumption of vanillate, an aromatic substrate degraded through PCA.^28^ The VanK transporter plays an overlapping role with PcaK in the uptake of 4HB.^36^ To test the role of these *van* genes, new strains with specific engineered deletions were derived from a parent that is 4HB^+^ on agar but 4HB^–^ in liquid (ACN2557). In these experiments (Figure S5), the transporter gene (*vanK*) was deleted and replaced with a drug cassette. This insertion most likely also prevents expression of the adjacent porin gene, *vanP*. This change was sufficient to confer growth in liquid medium with 4HB as the carbon source, suggesting that reduced 4HB uptake can limit the PobA-mediated production of PCA. A similar result can be achieved by the deletion of *pobA* or *pobR*, which may explain the basis of different requirements for *pobA*/*pobR* deletion in strains with and without *vanK­(P)*. In the absence of VanK (and VanP), lower levels of 4HB uptake could reduce the selective pressure for mutations in *pobA* or *pobR* to lower PCA production. The impact of *vanK* and *pobA* mutation is greater in liquid culture than on solid medium, consistent with these growth conditions being significantly different. Our results highlight the subtle nature of metabolic imbalances that can control the threshold for growth.

### Concluding Remarks

The prospect of applying *A. baylyi* for biotechnology derives both from its metabolic capabilities and extreme genetic malleability. Here, the successful introduction and optimization of a *meta*-cleavage pathway demonstrate that major metabolic changes can be orchestrated using *A. baylyi’s* natural transformation, gene amplification, and adaptive laboratory evolution. ADP1 is a promising strain to convert complex lignin-derived mixtures to chemical products.^5^ Nevertheless, *A. baylyi* remains limited in its ability to degrade some of the compounds derived from syringyl- and guaiacyl-lignin.^19^ The PCA 2,3-cleavage pathway provides new metabolic entry points for further expansion of the metabolism of nonnative lignin-derived substrates by offering different metabolic connections. Known pathways from other organisms can be similarly recapitulated in this host, and synthetic pathways can be designed, introduced, and optimized. The simultaneous degradation of multiple diverse aromatic compounds lies at the heart of strategies to maximize lignin valorization.^2,3,5^ With techniques used here, it may be possible to develop *A. baylyi* strains with metabolic capabilities that match lignin feedstocks. Recent studies suggest that *A. baylyi* strains with different capabilities may be mixed in consortia that augment the capabilities of individual isolates.^25^

*A. baylyi* has an atypical nonconvergent β-ketoadipate pathway. There are two branches of this pathway that funnel diverse compounds through one of two ring-cleavage substrates, catechol or PCA. In most bacteria these pathways converge.^48^ However, *A. baylyi* maintains parallel branches, each of which produces succinate and acetyl-CoA.^19^ Thus, in the newly engineered strains with the PCA *ortho*-cleavage pathway replaced by a *meta*-cleavage pathway, an intact *ortho*-cleavage pathway remains for compounds that are degraded, or could be degraded, through catechol. The effects have yet to be determined for the impact of these novel metabolic combinations on the metabolism of lignin-derived mixtures. Similarly, the impact of redirecting carbon through different pathways for bioproduction, which has been demonstrated in *P. putida*,^22^ has yet to be studied for these *A. baylyi* strains.

The CEMENT technique was developed to reveal critical combinations of mutations underlying complex physiological interactions. This method for identifying combinations of beneficial mutations is significantly simpler and faster than traditional methods of reverse engineering. PCR products amplified directly from mutants can be used as donor DNA. However, the full range of utility has yet to be demonstrated. For example, we do not know the upper limit of the total number of fragments that will result in a positive outcome. CEMENT can complement other methods that harness the power of natural transformation in *A. baylyi*.^26^ A related method of mutational analysis was developed wherein DNA fragments that confer benefits are selected and monitored during growth using a plate reader.^27^ This method, termed RAMSES (Rapid Advantageous Mutation Screening and Selection) was used to determine mutations that contribute to improved tolerance to a toxic compound. These rapid transformation methods help to direct subsequent investigations. Moreover, EASy-based methodology generates whole genome sequence data that can be coordinated with data-driven approaches to clarify the contributions of mutations that occur during adaptation.^49^

In the present study, our attention was directed toward a small set of mutations that enable growth of *A. baylyi* on 4HB using the foreign PCA 2,3-cleavage pathway. These mutations provide insight into aromatic compound metabolism that will guide future research. Moreover, this work lays the foundation to derive and optimize modular pathways for biotechnology using a methodology that mimics an accelerated evolutionary process. Some of the information gained, and the enzymes and pathways generated in *A. baylyi*, should be portable, with relevance to other industrially important microbes.

## Materials and Methods

### Media and Growth Conditions

*A. baylyi* cultures were grown in minimal medium (MM) with a carbon source, such as pyruvate (at 20 mM final concentration) or an aromatic compound (e.g., 4HB or PCA, at a final concentration in the range of 1–5 mM).^31,50^ *E. coli* cultures were grown in lysogeny broth (LB), also known as Luria–Bertani medium (10 g of Bacto-tryptone, 5 g of yeast extract, and 10 g of NaCl per L).^51^ Antibiotics were added as needed at final concentrations of 25 μg/mL for kanamycin (Km), 12.5 μg/mL each for streptomycin (Sm) and spectinomycin (Sp), and 150 μg/mL for ampicillin (Ap). To select multiple chromosomal copies of the Km-resistance marker, a final concentration of 1 mg/mL was used of Km. *E. coli* cultures and plates were incubated at 37 °C. *A. baylyi* cultures and plates were incubated at 37 or 30 °C. Liquid cultures were usually grown in 15 mL test tubes, aerated by shaking at 250 rpm.

For monitoring growth in a Synergy 1 plate reader (BioTek), 2 μL of an inoculum from a liquid culture grown ≥18 h were added to 198 μL of medium in the well of a Costar 3603 96-well plate. Plates were shaken at 282 rpm with a 3 mm orbit and incubated at 30 °C. OD_595_ readings were recorded every 30 min, with 8 measurements per well. Strains grew approximately 3-fold slower in the plate reader than in test tubes, but the relative growth rates between different strains were consistent regardless of growth method.

### Strains, Plasmids, and PCR Primers

*A. baylyi* strains with ACN designations (Table 1) were derived from the wild type, ADP1.^52,53^ Strains were constructed, as detailed in Table S1, using previously described methods.^9,26^ Briefly, a recipient strain was transformed by linear DNA carrying mutations. Donor DNA included restriction enzyme-digested plasmids, PCR fragments, and cell-free genomic DNA. New strains resulting from allelic replacement were selected by drug resistance or growth characteristics. When selection was not possible, strains were screened for phenotypic or genetic changes. In some cases, spontaneous mutants were directly selected by inoculating a parent strain on selective medium, such as 2 mM 4HB, and obtaining colonies. New strains were streak purified, and genotypes were confirmed by PCR and DNA sequence analysis.

*E. coli* strains XLI-Blue (Agilent Technologies) and DH5α^54^ were used as plasmid hosts. Plasmids and primers are listed in Tables S2 and S3, respectively. Omega cassettes were used to introduce drug resistance (ΩK confers Km^R^ and ΩS confers Sm^R^ and Sp^R^).^34,55^ In some cases, a selectable/counter-selectable *sacB* marker was employed.^56^

Standard molecular biology methods were used.^51^ For PCR, high-fidelity polymerases were used such as Q5 (NEB), PrimeSTAR MAX (Takara Biosciences), or Phusion (New England Biosciences). Plasmids were constructed by restriction digestion and ligation (Quick Ligation kit; New England Biolabs), with the NEBuilder kit (New England Biolabs), or by overlapping sequence assembly.^57^ Plasmids were confirmed by restriction digestion and local or whole-plasmid sequencing (Eurofins Genomics). Site-directed mutagenesis of plasmid DNA was conducted with mutagenic primers and methods based on the QuickChange II protocol.^58,59^ When PCR products were used in plasmid assembly or to transform *A. baylyi*, they were treated with the restriction enzyme DpnI (New England Biolabs) to degrade remaining template DNA.

To retrieve large chromosomal DNA segments in the *pra* gene region on plasmids, the gap-repair method was used.^11,37^ In this process, a recipient is transformed by a linearized plasmid that becomes circularized in vivo by homologous recombination. A chromosomal region is thereby captured on the plasmid, with the end points of the region determined by the sequences on the linear DNA that mediate recombination with the chromosome. One plasmid, pBAC1396, captured DNA inserted in Site 1, and another plasmid, pBAC1399, captured DNA inserted in Site 2. When used in these studies, each plasmid was linearized by SpeI. After 6 to 8 h of growth with linearized plasmids, *A. baylyi* transformants were plated to LB medium with ampicillin to select cells carrying the circularized plasmid. After overnight growth, Ap^R^ colonies were pooled, and plasmid DNA was extracted. Plasmids were used to transform *E. coli* where they are stable and can be isolated for further characterization.

### Genes, Locus Tags, Genome Sequencing, and Sequence Analysis

Chromosomal positions and locus tags correspond to those for the wild type, *A. baylyi* ADP1 (NCBI reference sequence NC_005966.1). To avoid confusion caused by differences in gene designations in various publications and databases, locus tags are provided for some of the genes in this study: *pobS* (ACIAD_RS07915), *quiA* (ACIAD_RS07910), *pobA* (ACIAD_RS07925), *gacA* (ACIAD_RS01230), *gacS* (ACIAD_RS13890), and *csrA* (ACIAD_RS05760). The *Paenibacillus pra* genes^29^ (GenBank accession number AB505864.1) were inserted in the chromosome at Site 1 or Site 2, as indicated in Figure 2 and S2.

Genomic DNA was sent to SeqCenter for Illumina whole-genome sequencing. Results were analyzed using Breseq on the UGA GACRC High-Performance Computing Cluster.^60,61^ In some cases, Geneious software was used.^62^ Localized DNA sequencing was done through Eton Biosciences and Eurofins, and the data analyzed using the SnapGene software (from Dotmatics; available at snapgene.com). Whole plasmid sequencing was done through Plasmidsaurus or Eurofins, and the data analyzed using the Snapgene software. Further analyses were conducted using Biocyc databases and pathway tools.^63,64^ Information and online tools were also used on Web sites for Uniprot and NCBI.^65,66^

### *A. baylyi* Transformation Assays (CEMENT)

Transformation methods for naturally competent *A. baylyi* have been described.^26^ For CEMENT assays with multiple donor DNA fragments, a recipient *A. baylyi* strain was grown overnight in 5 mL of MM with 20 mM pyruvate. The following day, 20 mM pyruvate was added to the 5 mL culture and allowed to grow with aeration for an additional 30–180 min. This culture, split between four 1.5 mL microfuge tubes, was pelleted at 5000 × G and the supernatant fluid removed. The four pellets were combined and washed with 1 mL MM. The cells were pelleted again, and 900 μL of MM were removed. The pellet was then suspended in the remaining 100 μL MM. This concentrated and washed culture was spread on selective medium on which the recipient strain could not grow. Approximately equimolar amounts of linearized plasmid DNA (100 ng–500 ng per fragment) was dropped onto the recipient strain shortly after the recipient culture had been spread and absorbed on the plate. After addition of the DNA, the plate was incubated, usually for three to 7 days.

### EASy Method

Procedures are detailed in an EASy method review.^10^ Briefly, parent strains constructed with the *pra*-genes in the chromosome were transformed with a linear plasmid carrying the SBF (Figures 2, 3 and S2). Linear SBF DNA was generated by restriction digestion of pBAC1415 (for Site 1) or pBAC1414 (for Site 2). Transformants were initially selected on plates with a Km concentration (1 mg/mL) that requires resistant cells to maintain multiple copies of the ΩK cassette adjacent to the *pra* genes on the amplicon. To confirm chromosomal duplication/amplification, PCR was used to detect the novel junction generated by the SBF, a genetic configuration that would not be present in the parent strain without a duplication. A primer set was used that was specific for Site 1 or Site 2: oMTV592 with oMTV669 or oMTV667 with oMTV668 (Table S3), respectively. Once a region of the chromosome was amplified, the culture was considered a mixed population, even when derived from a clonal colony, because copy number variation can occur stochastically.

Antibiotic selection was removed, and cells with genomic duplication/amplification were transferred to MM plates or cultures supplemented with 4HB or PCA as a sole carbon source. Constant selective pressure for growth on a single carbon source was maintained at 37 °C, with shaking. Cultures were diluted 1:100 in each daily subculture. The average copy number of the pra genes in each culture was tracked weekly using qPCR, as described below.

### qPCR Copy Number Analysis

To assess copy number during EASy experiments, qPCR was used to compare a region on the amplicon to one outside the amplicon (in *rpoA*) that is presumed to be in single copy.^10^ Primers (oMTV680 and oMTV681) were used to detect a 52 bp fragment (F52) in *praA*, the first gene of the *pra* operon, which is on the amplicon (Figure 3). Primers (oMTV274 and oMTV275, Table S3) were used for *rpoA*. Standard curves used genomic DNA from a strain (ACN1829) with a single copy of both *praA* and the control gene, *rpoA*. Standards were generated by making by making dilutions of the genomic DNA with final concentrations of: 5 ng/μL, 1 ng/μL, 0.2 ng/μL, 0.04 ng/μL and 0.008 ng/μL. Copy number was estimated by the ratio of F52 and *rpoA* dosage. qPCR data were acquired using the StepOnePlus Real-Time PCR System from Applied Biosystems. Runs were set up using the StepOneTM Software V2.3, and raw data were analyzed using the Run and Analyze Tool of software. An example of data, in Figure S3, illustrates standard curves and representative qPCR efficiency, which typically ranged from 90 to 100%.

### Testing for Growth Differences on Solid and Liquid 4HB Media

Some strains, such as ACN2557, grew on 4HB as the carbon source, at a final concentration of 2–5 mM on agar plates. A 4HB^+^ phenotype was defined as the ability to form colonies within 2–3 days when incubated at 37 or 30 °C. However, growth was not observed when 4HB+ cells were used to inoculate liquid cultures. Different growth conditions were evaluated. For comparisons, a standard protocol was followed for inoculation, culture conditions, and measurements. Strains were streaked on plates with pyruvate as the carbon source, and after small, uniform colonies were visible, cells were scraped and suspended in100 μL MM (no carbon source). Approximately 20 μL of this cell suspension was used to inoculate a tube with 2.7 mL of 5 mM 4HB MM. Cultures were grown at 37 °C with aeration, and samples were removed for OD_595_ measurements periodically during several days of incubation. Cultures that did not reach an OD_595_ of 0.1 in 3 days were designated 4HB^–^. Typically, cultures that were 4HB^+^ reached an OD_595_ of 0.2–0.5 in the same time frame. Some of the 4HB^–^ cultures were incubated for 5 days or more and still failed to reach the cutoff value (0.1). Results shown in Table S4 represent reproducible observations for all strains grown at least three times in duplicate.

## Supplementary Material



## Data Availability

Experimental data are presented in the publication and in the Supporting Information. Raw genome sequence data may be accessed from the NCBI Sequence Read Archive (SRA) using the following BioProject accession number: PRJNA1289738.

## Notes

Supporting figures: S1, Strain lineages; S2, Chromosomal insertion of foreign pra genes in the *A. baylyi* chromosome; S3, Representative qPCR data; S4, Growth rates of mutants using 4HB via the non-native PCA 2,3-cleavage pathway; S5, Effect of *vanK* deletion on growth in liquid culture using 4HB as the carbon source; S6, Integration of hydroxylase genes in two loci. Supporting tables: S1, strain information; S2, plasmid information; S3, oligo information; S4, Mutations in 4HB+ mutant ACN1936; S5, Mutations in *praC*; S6, Mutations in 4HB^+^ strains transformed by different combinations of donor DNA in CEMENT assays. Figures and tables in one file (PDF)

## ∥

Oak Ridge National Laboratory, Biosciences Division, Oak Ridge, Tennessee 37830, United States

## ⊥

Mayo Clinic, Internal Medicine, Baldwin Building, Rochester, Minnesota 55905, United States.

## #

Department of Microbial Infection and Immunity, The Ohio State University, Columbus, Ohio 43210, United States.
