# Supplementary material for: Rewiring Aromatic Compound Consumption: Chromosomal Amplification and Evolution of a Foreign Pathway in Acinetobacter baylyi ADP1
Source: ACS Synth Biol. 2025 Aug 27;14(9):3543–56. doi: 10.1021/acssynbio.5c00341 (PMC12455638; doi:10.1021/acssynbio.5c00341)
Supplement: Supplementary file 1 [file sb5c00341_si_001.pdf]

SUPPORTING INFORMATION

A.C. Baugh, M.P. Tumen-Velasquez, et al. ACS Synthetic Biology, 2025
DOI: 10.1021/acssynbio.5c00341

Rewiring Aromatic Compound Consumption: Chromosomal Amplification and Evolution of a Foreign Pathway in *Acinetobacter baylyi* ADP1

Author List

Alyssa C. Baugh,[1,#,a] Melissa P. Tumen-Velasquez,[1,#,a] Isabella R. Zempel,[1] Chantel V. Duscent-Maitland, [1] Lauren E. Slarks,[1, b] Justin B. Defalco,[1, c] Christopher W. Johnson,[2] Gregg T. Beckham,[2] and Ellen L. Neidle,[1, *]

[#] A.C.B. and M.P.T.-V. contributed equally *email: eneidle@uga.edu

[1]Department of Microbiology, University of Georgia, Athens, GA, USA

[2] Renewable Resources and Enabling Sciences Center, National Renewable Energy Laboratory, Golden, CO, 80401 USA

Present addresses:

[a]Oak Ridge National Laboratory, Biosciences Division, Oak Ridge, TN, 37830, USA

[b]Mayo Clinic, Internal Medicine, Baldwin Building, Rochester, MN, 55905, USA

[c]Department of Microbial Infection and Immunity, The Ohio State University, Columbus, OH, 43210 USA

Contents **Page**

Figure S1. Strain lineages S2

Figure S2. Chromosomal insertion of foreign *pra* genes in the *A. baylyi* chromosome S3

Figure S3. Representative qPCR data S4

Figure S4. Growth rates of mutants using the non-native PCA 2,3-cleavage pathway S5

Figure S5. Effect of *vanK* deletion on growth in liquid culture S6

Figure S6. Integration of hydroxylase genes in two loci S7

Table S1. *Acinetobacter baylyi* strains S8-14

Table S2. Plasmids S14-17

Table S3. Oligos S18-22

Table S4. Mutations in a 4HB$^+$ mutant, ACN1936 S23

Table S5. Mutations in *praC* S24

Table S6. Mutations after transformation in CEMENT assays S25

References for Supporting Information S26-27

DOI: 10.1021/acssynbio.5c00341

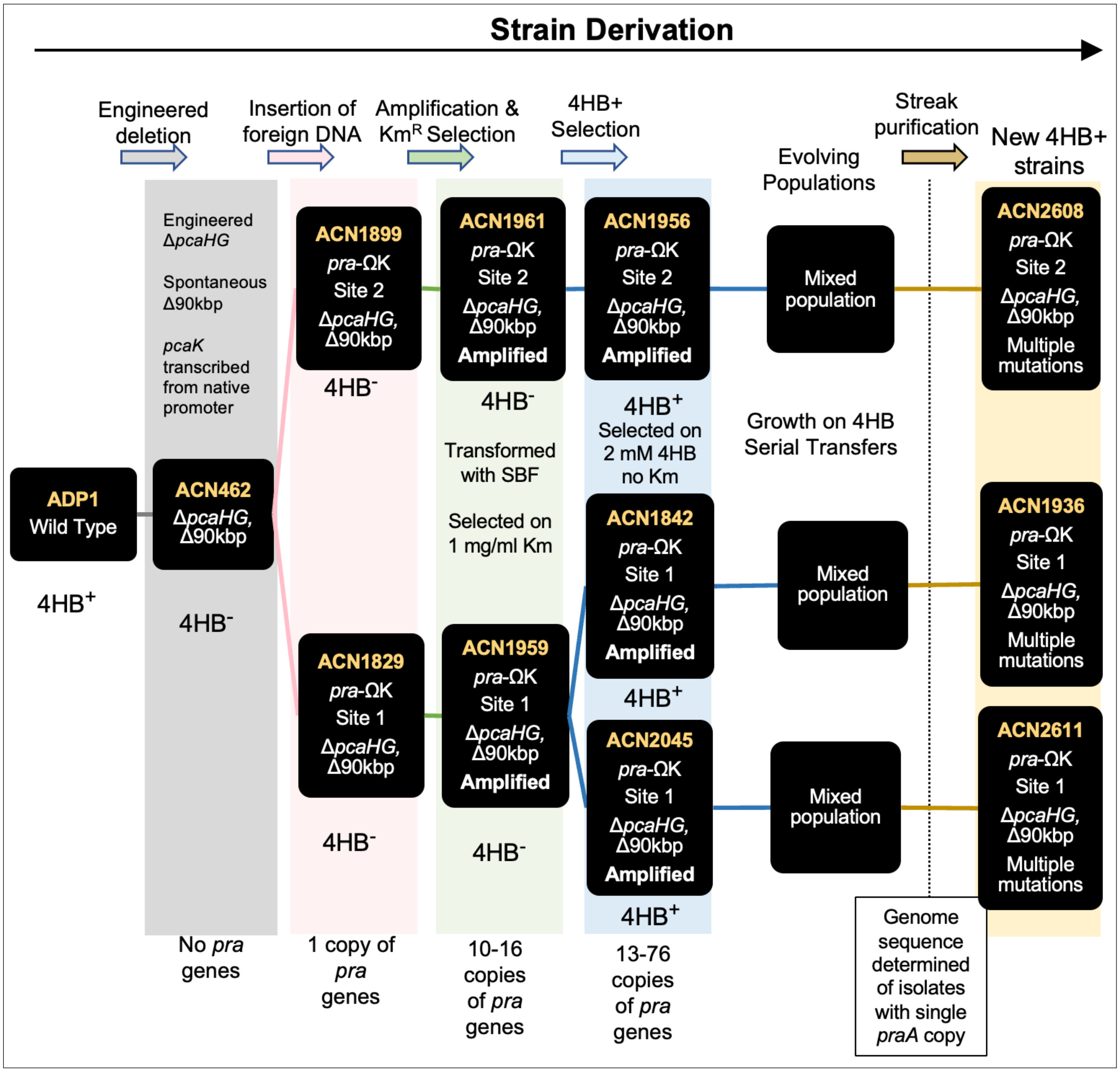

Figure S1. Strain lineages**.** All strains were derived from ADP1. Strain construction is detailed further in the main text and in Tables S1-S3.

DOI: 10.1021/acssynbio.5c00341

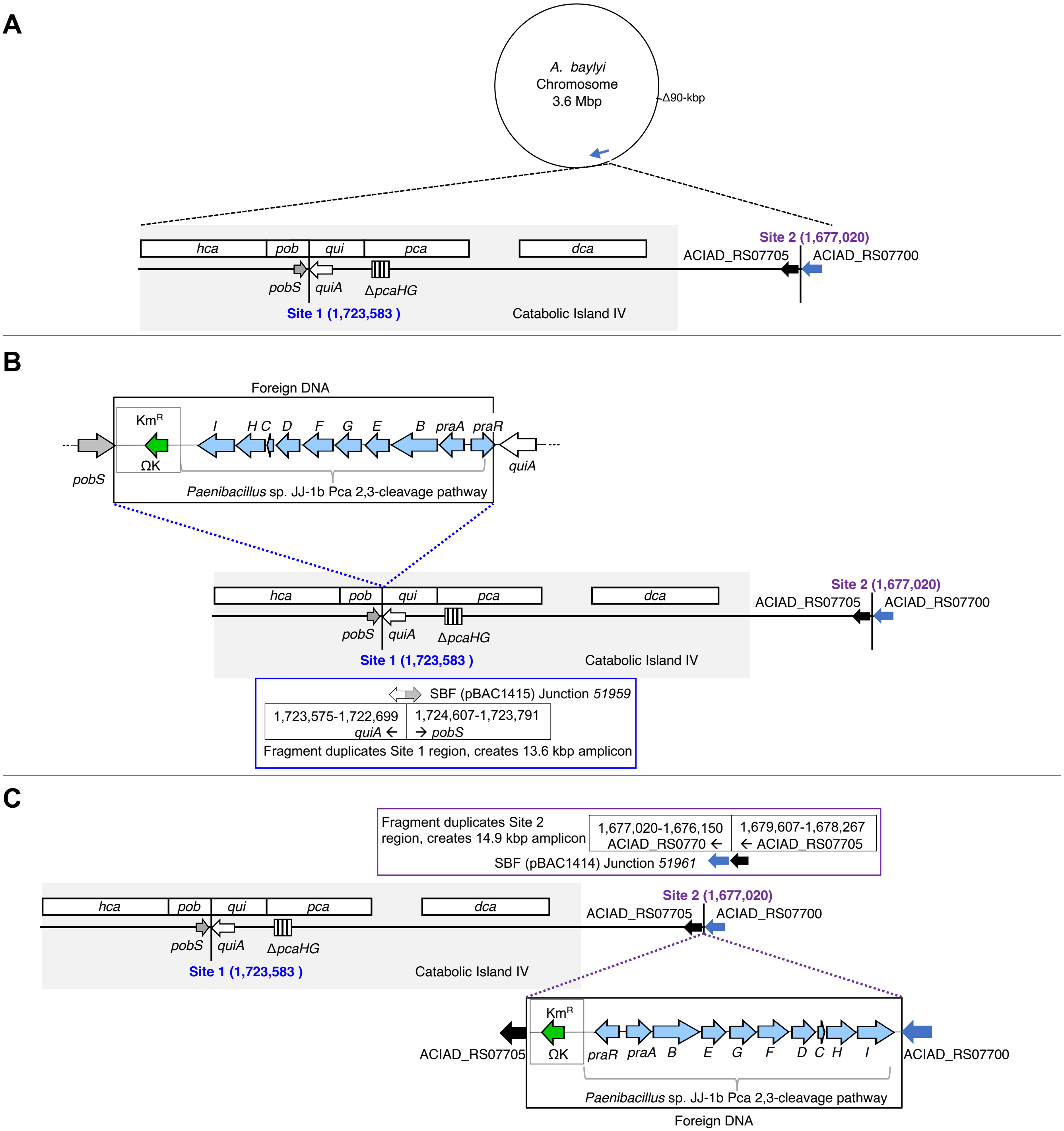

Figure S2. Chromosomal insertion of foreign *pra* genes in the *Acinetobacter baylyi* ADP1 chromosome. (A) Foreign genes were inserted in the chromosome in or near a cluster of genes involved in aromatic compound degradation (catabolic island IV, gray). The positions of gene groups in this region are drawn to scale. Numbers correspond to chromosomal positions (NCBI entry NC_005966). (B) Details concerning the integration and amplification of the foreign genes in Site 1. C) Details concerning the integration and amplification of the foreign genes in Site 2.

DOI: 10.1021/acssynbio.5c00341

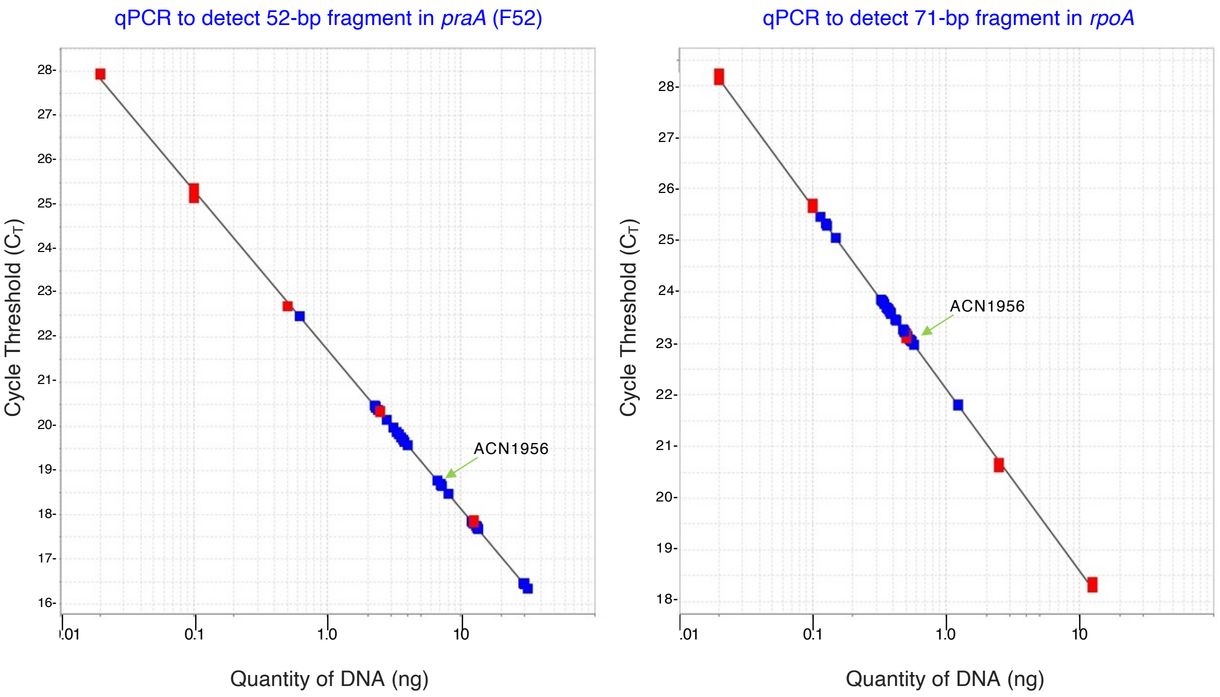

Figure S3. Representative qPCR data. To determine gene dosage of the amplified *pra*-gene region, the quantity of a 52-bp PCR fragment in *praA* (F52) was compared to that of a 71-bp PCR fragment in *rpoA* that was generated using the same template DNA in both reactions. Standard curves (red values) were generated from dilutions of ACN1829 genomic DNA (at concentrations of 5 ng/µl, 1 ng/µl, 0.2 ng/µl, 0.04 ng/µl and 0.008 ng/µl). After genomic DNA was isolated from 9 samples to use as template, two duplicate PCRs were evaluated for both reactions (blue values). For example, the green arrows point towards values associated with a sample of the evolving ACN1956 population. The arrows correspond to a ratio (copy number of F52) equal to 14 (7 ng/0.5 ng). For these data, the qPCR efficiencies for *praA* (F52) and *rpoA* were 90% and 92%, respectively. Typically, qPCR efficiencies were in the range of 90-100%. The primers used were oMTV680 and oMTV681 for *praA* (F52) and oMTV274 and oMTV275 for *rpoA*, Table S3.

DOI: 10.1021/acssynbio.5c00341

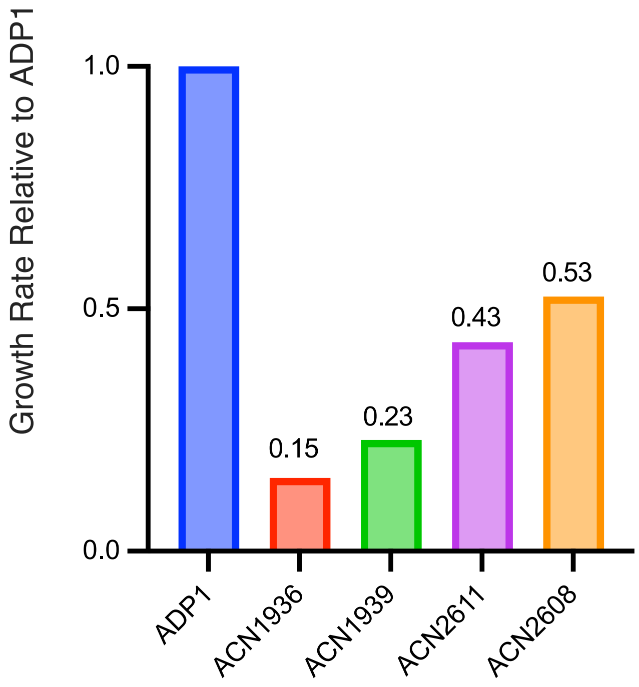

Figure S4. Growth rates of different strains on 2 mM 4HB as the sole carbon source relative to the wild type, ADP1. ADP1 grew on 4HB using the native PCA branch of the β-ketoadipate pathway with a generation time of 49 min. Strains ACN1936, ACN1939, ACN2611, and ACN2608, which all have a single copy of *praA,* grew using the nonnative PCA 2,3-cleavage pathway. The standard deviations for these cultures, grown in triplicate, were all within 15% of the value. These data are representative of different strains that were tested, all of which grew more slowly than the wild type, and all of which had different genomic mutations that were selected during adaptation (**Table S1**).

A

Strains derived by transformation of a recipient (ACN2557) that is 4HB$^-$ in liquid culture

New Strain	Donor DNA	Deletion introduced in chromosome of engineered strain	*vanK*	4HB$^+$ in liquid culture?
ACN3316	gDNA, ACN1829	Δ90-kbp; Δ 944,926-1,035,015	absent	yes
ACN3588	pBAC2207	Δ31 (A)-kbp; Δ974,866-1,005,605	present	no
ACN3589	pBAC2208	Δ31 (B)-kbp; Δ1,005,319-1,035,392	present	no
ACN3590	pBAC2217	Δ9-kbp; Δ964,809-974,249	absent	yes
ACN3591	pBAC2218	Δ14-kbp; Δ944,858-959,115	present	no
ACN3610	pBAC2245	Δ*vanK*::Gm^R-*sacB*	absent	yes

B

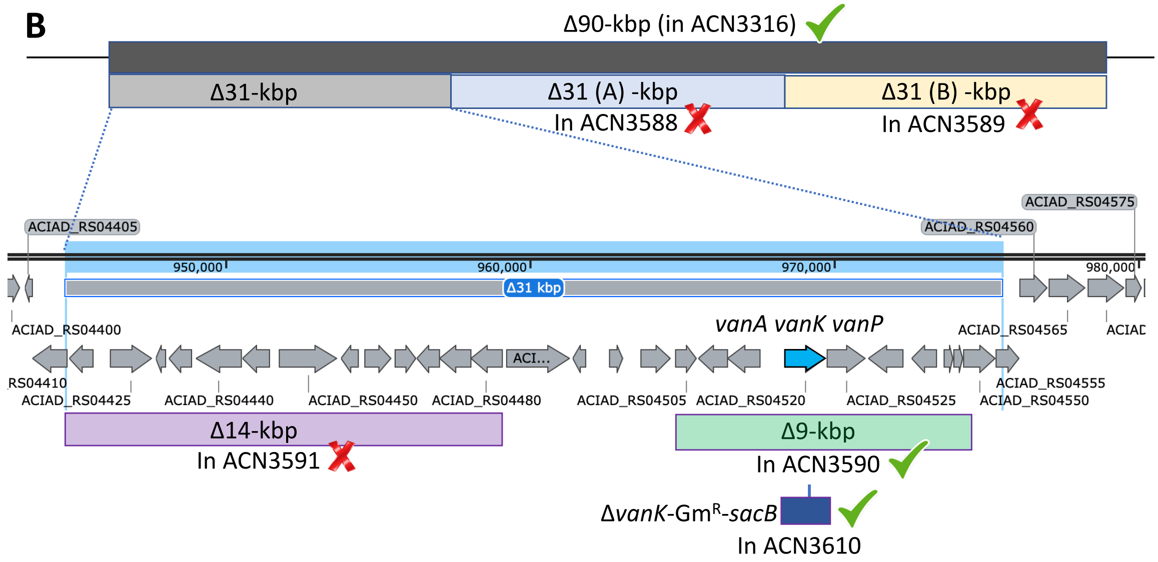

Figure S5. Loss of *vanK* can affect growth on 4HB in liquid culture. A parent strain, ACN2557, is 4HB$^+$ on solid medium but 4HB$^-$ in liquid culture. This strain carries mutations in *pobA, praC*, and *gacA,* and it has a wild-type *vanK* region (**Table S1**). (A) New strains were engineered by transforming ACN2557 with linear donor DNA to replace the corresponding chromosomal region with a specific deletion. To generate ACN3316, genomic DNA from ACN1829 was used. ACN1829 has Δ90-kbp and is 4HB$^-$ under all conditions. To construct the other strains, linear donor DNA was generated by PCR using a plasmid as template. A 4HB$^-$ designation indicates failure to reach an OD_{595} of at least 0.1 in liquid medium with 4HB as the carbon source in three days (see methods). (B) Diagram of chromosomal deletions. Introduction of Δ90-kbp (in ACN2557 to generate ACN3316) conferred 4HB growth in liquid (green check). Of three smaller deletions in this region, each ~31 kbp, two failed to confer growth on 4HB to the resulting strains (ACN3588 and ACN3589, red X). Of two smaller deletions (in ACN3591 and ACN3590), only ACN3590 was 4HB$^+$ in liquid culture. To test the role of *vanK*, this gene was deleted and replaced by a Gm^R-*sacB* cassette in ACN3610. Removal of *vanK* in ACN2557 (to create ACN3610) was sufficient to confer the 4HB$^+$ phenotype in liquid culture. This vanK deletion and cassette insertion likely also prevents the VanP porin from being expressed.

DOI: 10.1021/acssynbio.5c00341

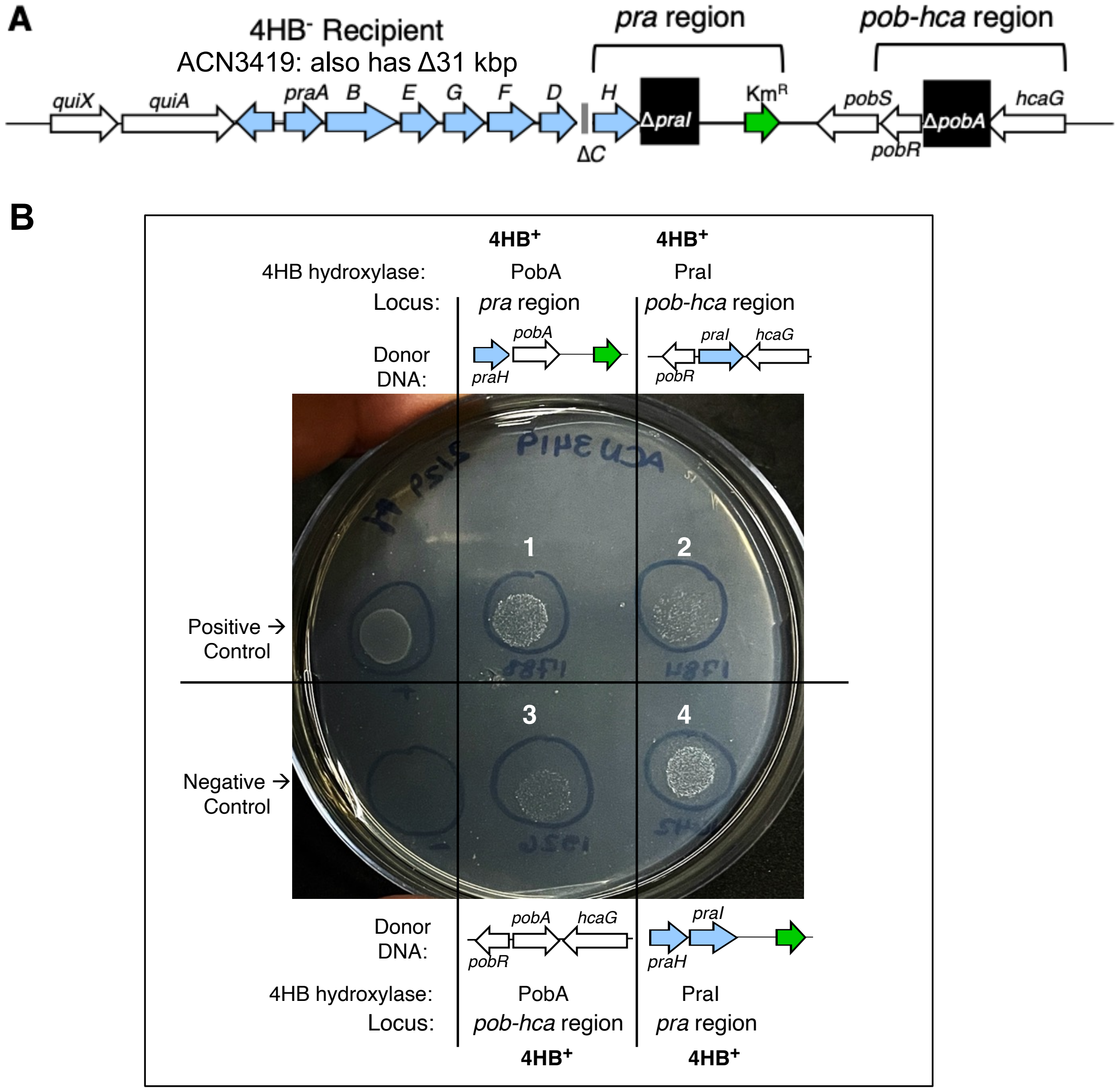

Figure S6. Integration of hydroxylase genes in two loci. This experiment differs only from that shown in **Figure 6** with respect to the recipient strain spread across the plate surface. The recipient strain in this experiment (ACN3419) is isogenic to that used previously (ACN2506) except that it additionally carries a chromosomal deletion (Δ31-kbp*53419*, **944,728 – 975,557).** This deletion is described further in **Table 2** and **Figure S5**. (A) Chromosomal region of the *praI* and *pobA* deletions that cause the recipient (ACN3419) to be 4HB$^-$. A 4HB$^+$ phenotype should result from expression of a functional 4HB hydroxylase because of the additional mutations in this strain (Δ*praC*, Δ*pca* genes, and disrupted *gacA*) (B) After this recipient (ACN3419) was spread on a 4HB plate, linear donor DNA fragments were dropped in spots. Allelic replacement should integrate *pobA* in its native locus (spot 2), *pobA* in place of *praI* (spot 3), *praI* in place of *pobA* (spot 5), and *praI* in place of its deletion (spot 6). Spots 1 and 4 were negative controls. Colonies arise where transformation by the donor DNA confers a 4HB$^+$ phenotype. Differences in the experimental results using the two different recipient strains are discussed in the main text.

SUPPORTING INFORMATION
A.C. Baugh, M.P. Tumen-Velasquez, et al**.** ACS Synthetic Biology, 2025
DOI: 10.1021/acssynbio.5c00341

Table S1. *Acinetobacter baylyi* strains[a,b,c]

**Strain**	**Relevant Characteristics**	**Source**
ADP1	Wild type strain (BD413); 4HB$^+$	1
ACN462	Δ*pcaHG5462,* Δ90kbp*5462;* 4HB$^-$<br>Engineered deletion of PCA 3,4-dioxygenase genes (*pcaHG)* Δ90kbp*5462=* 90,164-bp spontaneous chromosomal deletion (**944,926-1,035,015**) discovered in this study by whole genome sequencing.	2
ACN1829	Δ*pcaHG5462,* Δ90kbp*5462, praRABEGFDCHI*-ΩK*51829;* 4HB$^-$<br>Insertion of *pra* genes from *Paenibacillus* sp. JJ-1b[3] in Site 1 (Figure 2) downstream of *quiA* (**1,723,583**)<br>pBAC1413/XmnI × ACN462[d]	This study
ACN1842	Δ*pcaHG5462,* Δ90kbp*5462*, *praRABEGFDCHI*-ΩK*51829,* Amplified population (junction *SBF51959);* 4HB$^+$<br>Multiple chromosomal copies of a 13.6-kbp amplicon including *praRABEGFDCHI* and ΩK (inserted in Site 1); duplication connects **1,722,699** in ACIAD_RS07910 (*quiA*) and **1,724,607** in ACIAD_RS07915 (*pobS*). Duplication junction was introduced in ACN1959, by the SBF carried on pBAC1415, and amplification was selected by high-level Km^R. ACN1842 (4HB$^+$) was derived by direct selection of ACN1959 (4HB$^-$) on solid 4HB medium	This study
ACN1899	Δ*pcaHG5462*, Δ90kbp*5462*, ΩK-*praRABEGFDCHI51899;* 4HB$^-$<br>Insertion of *pra* genes from *Paenibacillus* sp. JJ-1b[3] in Site 2 (**Figures 2 & S2**) downstream of ACIAD_RS07700 (**1,677,020)**<br>pBAC1486/XmnI × ACN462[d]	This study
ACN1936	Δ*pcaHG5462*, Δ90kbp*5462, praRABEGFD(C)HI*-ΩK*51936, praC51936,* Δ*pobS-hcaC51936, rpoB51936, pbpA51936,* ACIAD_RS01270 51936*,* ACIAD_RS06205 51936*;* 4HB$^+$<br>Single copy of a 13.6-kbp amplicon including *pra*-genes and ΩK in Site 1. Mutations arose during adaptive laboratory evolution (see **Table S4**) EASy-derived 4HB$^+$ isolate from evolving population of ACN1842	This study
ACN1937<br>ACN1938	See ACN1936. Three isolates were sequenced from the same population (ACN1936, ACN1937, and ACN1938). ACN1937 had all the mutations listed in **Table S4**. ACN1938 had no mutation in ACIAD_RS01270, but it had the other mutations listed in **Table S4**.	This study
ACN1939	Δ*pcaHG5462* Δ90kbp*5462, praRABEGFD(C)HI*-ΩK*51936, praC51936, pobRA51939,* additional mutations; 4HB$^+$<br>Engineered to incorporate the *pra* genes, with mutated *praC*, from ACN1936. Transformants (4HB$^-$) were selected on Km to integrate the *pra* region. Next, ACN1939 was obtained by selection on 4HB of the 4HB$^-$ parent. A 7 bp deletion of AAAATAG, (**1,725,924-1,725,930**) affects the *pobA* and *pobR* operator-promoter region<br>pBAC1536/SnaBI × ACN462[d]	This study

ACN1956	Δ*pcaHG5462,* Δ90kbp*5462,* ΩK-*praRABEGFDCHI51899*, amplified population (has *SBF51961* junction); $4HB^+$<br>Multiple chromosomal copies of a 14.9-kbp amplicon including *praRABEGFDCHI* and ΩK; duplication connects **1,679,399** (in ACIAD_RS07705) and **1,676,149** (in ACIAD_RS07700), Site 2. Duplication junction was introduced in ACN1961, by the SBF carried on pBAC1414. ACN1956 ($4HB^+$) was derived by direct selection of ACN1961 ($4HB^-$) on solid 4HB medium	This study
ACN1959	Δ*pcaHG5462,* Δ90kbp*5462, praRABEGFDCHI*-ΩK*51829*, amplified population (has *SBF51959* junction); $4HB^-$<br>Multiple chromosomal copies of a 13.6-kbp amplicon including *praRABEGFDCHI* and ΩK; precise chromosomal duplication connects **1,722,699** (in ACIAD_RS07910, *quiA*) and **1,724,607** (in ACIAD_RS07915, *pobS*) Site 1, selected on 1 mg/ml Km (ACN1959 population is $4HB^-$)<br>pBAC1415/AatII × ACN1829[d]	This study
ACN1961	Δ*pcaHG5462,* Δ90kbp*5462,* ΩK-*praRABEGFDCHI51899*, amplified population (has *SBF51961* junction); $4HB^+$<br>Multiple chromosomal copies of a 14.9-kbp amplicon including *praRABEGFDCHI* and ΩK (in Site 2, **Figures 2 & S2**), chromosomal duplication connects **1,679,399** (in ACIAD_RS07705) and **1,676,149** (in ACIAD_RS07700), selected on 1 mg/ml Km. ACN1961 population is $4HB^-$<br>pBAC1414/AatII × ACN1899[d]	
ACN1992	Δ*pcaHG5462* Δ90kbp*5462, praRABEGFD(C)HI*-ΩK*51936, praC51936, pobR51992,* additional mutations; $4HB^+$<br>Engineered to incorporate the *pra* genes in Site 1, with mutated *praC*, from ACN1936. Transformants ($4HB^-$) were selected on Km to integrate the *pra* region. Next, ACN1992 was obtained by direct selection on 4HB of a $4HB^-$ transformant. The *pobR* gene was disrupted by an IS*1236* insertion. The insertion disrupts the CDS after the sequence CCT encoding P258. The insertion, adding 1237 bp after **1,725,043** causes a frameshift predicted to alter the C-terminus of PobR and render the regulatory protein inactive.<br>pBAC1536/SnaBI × ACN462[d]	This study
ACN1993	Δ*pcaHG5462* Δ90kbp*5462, praRABEGFD(C)HI*-ΩK*51936, praC51936, pobA51993,* additional mutations; $4HB^+$<br>Engineered to incorporate the *pra* genes, with mutated *praC*, from ACN1936. Isolated by direct selection on 4HB of its $4HB^-$ Km^R parent that was obtained by transformation and selection for drug resistance. A 149-bp deletion (1,726,708-1,726,856) inactivates *pobA*<br>pBAC1536/SnaBI × ACN462[d]	This study
ACN2045	Δ*pcaGH5462*, Δ90kbp*5462*, *praRABEGFDCHI*-ΩK*51829*, Amplified population (junction *SBF51959;* $4HB^+$<br>Multiple chromosomal copies of a 13.6-kbp amplicon including *praRABEGFDCHI* and ΩK; duplication connects **1,722,699** in ACIAD_RS07910 (*quiA*) and **1,724,607** in ACIAD_RS07915 (*pobS*). Duplication junction was introduced in ACN1959 by the SBF carried on	This study

	pBAC1415. ACN2045 (4HB^{+}) was derived by direct selection of ACN1959 (4HB^{-}) on solid 4HB medium	
ACN2106	*pcaK52106,* Δpca*UIJFBDCHG52106;* 4HB^{-}<br>Deletion of *pca* genes (**1,707,706 – 1,713,981**) replaced with the *lac* promoter (P_{lac}) controlling *pcaK*<br>pBAC1561/AatII × ADP1[d]	This study
ACN2212	*pcaK52106, ΔpcaUIJFBDCHG52106, pobA::*ΩS*52212;* 4HB^{-}<br>Interruption of *pobA* by insertion of ΩS (**1,726,129**)<br>pBAC1584/AatII × ACN2106[d]	This study
ACN2235	*pcaK52106,* Δ*pcaUIJFBDCHG52106, pobA*::ΩS*52212* *praRABEGFD(C)HI*-ΩK*52611, praC52611;* 4HB^{-}<br>Insertion of *pra* genes from ACN2611 (carried on pBAC1643)<br>pBAC1643/SnaBI × ACN2212[d]	This study
ACN2237	*pcaK52106,* Δ*pcaUIJFBDCHG52106, pobA*::ΩS*52212*, *praRABEGFD(C)HI*-ΩK*52237,* Δ*praC52237;* 4HB^{-}<br>Insertion of *pra* genes (without *praC)* in Site 1(**1,723,583)**. In the *pra*-gene operon, 783 bp were deleted immediately after the stop signal (TAG) of *praD.* The RBS for *praH* is intact.<br>pBAC1642/SnaBI × ACN2212[d]	This study
ACN2271	*pcaK52106,* Δ*pcaUIJFBDCHG52106, pobA*::ΩS*52212* *praRABEGFD(C)HI*-ΩK*52611, praC52611, gacS52271;* 4HB^{+}<br>*gacS* (ACIAD_RS13890), at position 636 in the CDS (**2,999,101**), a deletion (T) shifts the frame after encoding $L_{111.}$ S_{112} (AGU) → S_{112} (AGC). Frameshift would cause termination after residue 263 instead of the 932 AA wild type GacS. Derived from ACN2235 by direct selection on a 4HB plate	This study
ACN2276	*pcaK52106,* Δ*pcaUIJFBDCHG52106, pobA*::ΩS*52212*, *praRABEGFD(C)HI*-ΩK*52237,* Δ*praC52237, gacA52276;* 4HB^{+}<br>*gacA (*ACIAD_RS01230), at position 112 in the CDS (**261,392**)<br>GAA→TAA truncates GacA after residue 37 (WT 211 residues)<br>Derived from ACN2237 by direct selection on a 4HB plate, no additional mutations aside from *gacA* were identified	This study
ACN2317	*pcaK52106,* Δ*pcaUIJFBDCHG52106, pobA*::ΩS*52212*, *praRABEGFD(C)HI*-ΩK*52237,* Δ*praC52237, gacA52276,* other mutations; 4HB^{+}<br>Derived from ACN2276 after serial transfers for 3 weeks to improve growth on 4HB	This study
ACN2401	P_{D5H6}-*pcaK52401,* Δ*pcaUIFJBDKCHG52401;* 4HB^{-}<br>Deletion of *pca* genes **(1,707,705 - 1,717,170)** replaced with the D5H6[4] promoter (P_{D5H6}) controlling *pcaK*<br>pBAC1717/AatII × ADP1[d]	[4]
ACN2416	P_{D5H6}-*pcaK52401,* Δ*pcaUIFJBDKCHG52401, hcaE*::*sacB*-ΩS*52416*, Δ*pobA52440;* 4HB^{-}<br>*sacB*-ΩS inserted adjacent to Δ*pobA* (**1,725,951 – 1,727,165**)	This study

	pBAC1585/AatII × ACN2401[d]	
ACN2440	P_{D5H6}-*pcaK52401, ΔpcaUIFJBDKCHG52401, ΔpobA52440;* 4HB$^-$<br>wild-type DNA in the *hca* region (replaces marker in *hcaE* of recipient strain ACN2416)<br>pBAC1578/AatII × ACN2416[d]	This study
ACN2449	P_{D5H6}-*pcaK52401, ΔpcaUIFJBDKCHG52401, ΔpobA52440, praRABEGFD(C)HI-*ΩK*52237, ΔpraC52237;* 4HB$^-$<br>Insertion of *pra* genes with Δ*praC* downstream of *quiA* (**1,723,583)**[d]<br>pBAC1642 /SnaBI × ACN2440[d]	This study
ACN2474	P_{D5H6}-*pcaK52401, ΔpcaUIFJBDKCHG52401, ΔpobA52440, gacA52276, sacB*-Ω*S52474;* 4HB$^-$<br>*sacB*-ΩS upstream of mutated *gacA52276* (inserted after **261,508**), used to introduce *gacA* mutation in the chromosome<br>pBAC1786/AatII × ACN2440[d]	This study
ACN2504	P_{D5H6}-*pcaK52401, ΔpcaUIFJBDKCHG52401, praRABEGFDCHI-*ΩK*51829;* 4HB$^-$<br>Insertion of *pra* genes downstream *quiA* (**1,723,583)** in Site 1, has wild-type DNA in the 90 kbp region<br>pBAC1413/SnaBI × ACN2401[d]	This study
ACN2506	P_{D5H6}-*pcaK52401, ΔpcaUIFJBDKCHG52401, ΔpobA52440, praRAB(C)EGFDH(I)-*ΩK*52506, ΔpraC52237, ΔpraI52506, gacA52276, sacB*-Ω*S52474;* 4HB$^-$<br>Insertion of *pra* genes with Δ*praC* and Δ*praI* downstream of *quiA* (**1,723,583)**<br>pBAC1783/SnaBI × ACN2474[d]	This study
ACN2517	P_{D5H6}-*pcaK52401, ΔpcaUIFJBDKCHG52401, praRABEGFD(C)HI-*ΩK*52237, ΔpraC52237 ΔpobA52440, gacA52276, sacB*-ΩS*52474*<br>*sacB*-ΩS upstream of mutated *gacA52276* (inserted after **261,508**), used to introduce *gacA* mutation in the chromosome<br>pBAC1786/AatII × ACN2449[d]	This study
ACN2557	P_{D5H6}-*pcaK52401, ΔpcaUIFJBDKCHG52401, ΔpobA52440, praRABEGFD(C)HI-*ΩK*52237, ΔpraC52237, gacA52276;* 4HB$^+$ on plates and 4HB$^-$ in liquid<br>DNA from plasmid replaced the counter-selectable marker (*sacB*-Ω*S52474),* retaining the adjacent *gacA52276*<br>pBAC1711/AatII × ACN2517[d]	This study
ACN2608	Δ*pcaHG5462,* Δ90kbp*5462,* ΩK-*praRABEGFD(C)HI52608, praC52608,* additional uncharacterized mutations; 4HB$^+$<br>Single copy of *praR-praA* region within an amplicon encompassing the *pra*-genes and ΩK. Frameshift mutation in *praC* (see **Table S5**). Rearrangements within the amplicon complicated further analysis. EASy-derived 4HB$^+$ isolate from evolving population of ACN1956	This study

ACN2611	Δ*pcaHG5462,* Δ90kbp*5462, praRABEGFD(C)HI*-ΩK*52611, praC52611,* additional uncharacterized mutations; $4HB^+$<br>Single copy of *praR-praA* region within an amplicon encompassing the *pra*-genes and ΩK. Frameshift mutation in *praC* (see **Table S5**). Rearrangements within the amplicon complicated further analysis. EASy-derived $4HB^+$ isolate from evolving population of ACN2045.	This study
ACN3315	Δ*pcaHG5462,* Δ90kbp*5462, praRAB(C)EGFDCHI*-ΩK*53315, praC53315,* Δ*gacA53315,* Δ*pobA52440, relA53315;* $4HB^+$<br>Deletions of *praC, gacA,* and/or *pobA* were used as donor DNA in CEMENT assays with ACN1829 as recipient. $4HB^+$ transformants were streak purified, named, and sequenced (see **Table S6**). ACN3315 was isolated after transformation with both Δ*gacA* and Δ*pobA* DNA.<br>pBAC2184/BamHI + NcoI (Δ*gacA53315*) and pBAC1578/EcoRI + EcoRV (Δ*pobA52440)* × ACN1829[d]	This study
ACN3316	Δ*pcaHG5462,* Δ90kbp*5462, praRABEGFD(C)HI*-ΩK*52237,* Δ*praC52237,* Δ*gacA53315,* Δ*pobA52440;* $4HB^+$<br>Deletions of *praC, gacA,* and/or *pobA* were used as donor DNA in CEMENT assays with ACN1829 as recipient. $4HB^+$ transformants were streak purified, named, and sequenced (see **Table S6**). ACN3316 was isolated after transformation with Δ*gacA,* Δ*praC,* and Δ*pobA* DNA.<br>pBAC2184/BamHI + NcoI (Δ*gacA53315*) and pBAC1578/EcoRI + EcoRV (Δ*pobA52440)* and pBAC1642/SnaBI + SalI (Δ*praC52237*) × ACN1829[d]	This study
ACN3317	Δ*pcaHG5462,* Δ90kbp*5462, praRABEGFD(C)HI*-ΩK*52237,* Δ*praC52237,* Δ*gacA53315;* $4HB^+$<br>Deletions of *praC, gacA,* and/or *pobA* were used as donor DNA in CEMENT assays with ACN1829 as recipient. $4HB^+$ transformants were streak purified, named, and sequenced (see **Table S6**). ACN3317 was isolated after transformation with both Δ*gacA* and Δ*praC* DNA.<br>pBAC2184/BamHI + NcoI (Δ*gacA53315*) and pBAC1642/SnaBI + SalI (Δ*praC52237*) × ACN1829[d]	This study
ACN3318	Δ*pcaHG5462,* Δ90kbp*5462, praRAB(C)EGFDCHI*-ΩK*52237* Δ*praC52237, gacS53318,* Δ*pobA52440, benK53318;* $4HB^+$<br>Deletions of *praC, gacA,* and/or *pobA* were used as donor DNA in CEMENT assays with ACN1829 as recipient. $4HB^+$ transformants were streak purified, named, and sequenced (see **Table S6**). ACN3318 was isolated after transformation with both Δ*pobA* and Δ*praC* DNA.<br>pBAC1578/EcoRI + EcoRV (Δ*pobA52440)* and pBAC1642/SnaBI + SalI (Δ*praC52237*) × ACN1829[d]	This study
ACN3326	P_{D5H6}-*pcaK52401,* Δ*pcaUIFJBDKCHG52401,* Δ*pobA52440, praRABEGFD(C)HI*-ΩK*52237,* Δ*praC52237, gacA52276,* Δ90kbp*5462;* $4HB^+$<br>Selection in 4HB liquid culture after transformation followed by streak purification of one colony; whole-genome sequencing revealed the only change in the transformant was acquisition of Δ90kbp*5462* from the donor DNA<br>ACN1829 DNA × ACN25557[d]	This study

ACN3370	P_{D5H6}-*pcaK52401, ΔpcaUIFJBDKCHG52401, ΔpobA52440, praRAB(C)EGFDH(I)*-ΩK*52506, ΔpraC52237, ΔpraI52506, ΔgacA53315;* 4HB$^-$<br>Deletion of *gacA* (**260,946 – 261,500)** from plasmid selected by replacement of adjacent *sacB* on chromosome of recipient<br>pBAC2184/AhdI × ACN2506[d]	This study
ACN3419	P_{D5H6}-*pcaK52401, ΔpcaUIFJBDKCHG52401, ΔpobA52440, praRAB(C)EGFDH(I)*-ΩK*52506, ΔpraC52237, ΔpraI52506, ΔgacA53315, Δ31kbp53419;* 4HB$^-$<br>ΩS replaces a 31-kbp fragment (**944,728 – 975,557,** see **Table 2**)<br>pBAC2220/AhdI × ACN3370[d]	This study
ACN3444	Δ*pcaHG5462,* Δ90kbp*5462, praRABEGFD(C)HI*-ΩK*52237,* Δ*praC52237,* Δ*gacA53315,* Δ*pobA52440,* Δ*csrA*::ΩS*53444;* 4HB$^-$<br>ΩS replaces *csrA* (**1,249,354 – 1,249,616**)<br>pBAC2183/AhdI & AatII × ACN3316[d]	This study
ACN3486	Δ*pcaHG5462,* Δ90kbp*5462, quiA*-ΩS*53486,* Δ*gacA53315,* Δ*pobA52440;* 4HB$^-$<br>No *pra* genes, Sp^R Sm^R resistance marker (ΩS) inserted in Site 1 of chromosome (removing *praRABEGFD(C)HI*-ΩK*52237,* Δ*praC52237* region of ACN3316).<br>pBAC2244/AhdI & AatII × ACN3316[d]	This study
ACN3496	Δ*pcaHG5462,* Δ90kbp*5462, praRABEGF(DC)HI*-ΩK*53496* Δ*praDC53496 gacA53315,* Δ*pobA52440;* 4HB$^-$<br>The *pra*-genes (without *praC* and *praD)* replaced *quiA*-ΩS*53486* in Site 1; the RBS of *praH* is intact.<br>pBAC1679/BsaI × ACN3486[d]	This study
ACN3514	Δ*pcaHG5462*<br>Genotype of transformant confirmed after identification in a screen for loss of ability to grow on 4HB<br>ACN462 PCR product × ADP1[d]	This study
ACN3515	Δ*pcaHG5462, praRABEGFDCHI*-ΩK*51829*<br>Genotype of transformant confirmed after selection for Km^R<br>pBAC1413/XmnI × ACN3514[d]	This study
ACN3588	P_{D5H6}-*pcaK52401,* Δ*pcaUIFJBDKCHG52401,* Δ*pobA52440, praRABEGFD(C)HI*-ΩK*52237,* Δ*praC52237, gacA52276,* Δ31(A)-kbp *53588;* deletion: **Δ974,866-1,005,605**; 4HB$^+$ on plates and 4HB$^-$ in liquid<br>PCR fragment from pBAC2207 × ACN2557[d] see **Figure S4**	This study
ACN3589	P_{D5H6}-*pcaK52401,* Δ*pcaUIFJBDKCHG52401,* Δ*pobA52440, praRABEGFD(C)HI*-ΩK*52237,* Δ*praC52237, gacA52276,* Δ31(B)-kbp *53589;* deletion: **Δ1,005,319-1,035,392**; 4HB$^+$ on plates and 4HB$^-$ in liquid<br>PCR fragment from pBAC2208 × ACN2557[d] see **Figure S4**	This study

DOI: 10.1021/acssynbio.5c00341

ACN3590	P_{D5H6}-*pcaK52401, ΔpcaUIFJBDKCHG52401, ΔpobA52440, praRABEGFD(C)HI*-ΩK*52237, ΔpraC52237, gacA52276,* Δ9-kbp *53590;* deletion: Δ**964,809-974,249**; 4HB$^+$ on plates and 4HB+ in liquid<br>PCR fragment from pBAC2217 × ACN2557[d] see **Figure S4**	This study
ACN3591	P_{D5H6}-*pcaK52401, ΔpcaUIFJBDKCHG52401, ΔpobA52440, praRABEGFD(C)HI*-ΩK*52237, ΔpraC52237, gacA52276,* Δ14-kbp *53591;* deletion: Δ**944,858-959,115**; 4HB$^+$ on plates and 4HB$^-$ in liquid<br>PCR fragment from pBAC2218 × ACN2557[d] see **Figure S4**	This study
ACN3610	P_{D5H6}-*pcaK52401, ΔpcaUIFJBDKCHG52401, ΔpobA52440, praRABEGFD(C)HI*-ΩK*52237, ΔpraC52237, gacA52276, ΔvanK*::Gm^R-*sacB 53510;* deletion: Δ*vanK* **968,383-969,729**; 4HB$^+$ on plates and 4HB+ in liquid<br>PCR fragment from pBAC2245 × ACN2557[d] see **Figure S4**	This study

[a] *A. baylyi* strains were all derived from the wild-type strain (ADP1), previously known as *Acinetobacter calcoaceticus* ADP1 or *Acinetobacter sp.* ADP1

[b]Abbreviations used in this table: Synthetic Bridging Fragment (SBF), omega cassette conferring Km^R (ΩK), omega cassette conferring Sm^{R}Sp^R (ΩS)

[c]Bold numbers correspond to positions on the ADP1 chromosome in NCBI entry NC_005966.

[d]For strains generated by allelic replacement, transformation is indicated by the donor DNA × recipient strain. Plasmids used as donor DNA were linearized by restriction digestion, indicated by plasmid name/restriction enzyme(s). When PCR products were used as donor DNA, they were first treated with DpnI to degrade the methylated template DNA. Plasmids and primers described further in Tables **S2** and **S3.** PCR and/or DNA sequencing were used to confirm the genotype of the final strain and to ensure that plasmids were not integrated via single recombination events.

Table S2. Plasmids[a]

**Plasmid**	**Relevant Characteristics**	**Source**
pRMJ1	Ap^{R}Km^R; source of *sacB*-ΩK	[5]
pUC18	Ap^R; cloning vector	[6]
pUC19	Ap^R; cloning vector	[6]
pMiniT	ApR; cloning vector	New England Biolabs (NEB)
pBRI23	Ap^R; source of the *Paenibacillus* JJ-1b PCA 2,3-cleavage pathway (*praRABEGFDCHI*)	[3], Gift from Prof. Eiji Masai
pUI1637	Ap^{R}Km^R; source of ΩK	[7]
pUI1638	Ap^{R}Sm^{R}Sp^R; source of ΩS	[7]
pBTL-2	Km^R; source of P_{lac} (*lac* promoter)	[8]
pBAC1384	Ap^R; *praRA*; pBRI123 digested with KpnI, fragment containing the vector backbone and *praR* and partial *praA* religated (removing *praBEGFDCHI*)	This study

pBAC1396	Ap^{R}Km^R; ADP1 DNA surrounding Site 1 (*quiA* and *pobS*) with ΩK; DNA added to this plasmid can be integrated in chromosomal Site 1	[4]
pBAC1397	Ap^R; ACIAD_RS07695, ACIAD_RS07700-ACIAD_RS07705; NEBuilder assembly of three PCR-generated fragments: (1) ACIAD_RS07695, ACIAD_RS07700 (ADP1 template with oMTV568 & oMTV609)[b], (2) ACIAD_RS07705 (ADP1 template with oMTV569 & oMTV608)[b] and (3) vector DNA (pUC18 template with oMTV570 & oMTV571)[b]. SacI site was introduced between ACIAD_RS07700 and ACIAD_RS07705; ADP1 DNA on either side of Site 2 for chromosomal integration	This study
pBAC1399	Ap^{R}Km^R; ACIAD_RS07700-ΩK-ACIAD_RS07705; ΩK from pUI1637 digested with SacI ligated to SacI-digested pBAC1397	This study
pBAC1406	Ap^R; *praRA*; NEBuilder assembly of two PCR-generated fragments: (1) *praRA* (pBRI23 template with oMTV610 & oMTV611)[b] and (2) vector DNA (pBAC1384 template with oMTV612 & oMTV613)[b]	This study
pBAC1408	Ap^R; *praRABEGFDCHI*; *praABEGFDCHI* from pBRI23 digested with KpnI and PspOMI ligated to KpnI and PspOMI-digested pBAC1406	This study
pBAC1413	Ap^{R}Km^R; *praRABEGFDCHI*-ΩK*51829* *pra* genes from pBAC1408 digested with SpeI and PspOMI and ligated into pBAC1396 (SpeI and PspOMI digested), allows foreign DNA (*pra*-genes and ΩK) to be integrated at Site 1	This study
pBAC1414	Ap^R; synthetic bridging fragment for ACIAD_RS07705 and ACIAD_RS07700 (Site 2); NEBuilder assembly of three PCR-generated fragments: (1) ACIAD_RS07705 (ADP1 template with oMTV661 & oMTV662)[b], (2) ACIAD_RS07700 (ADP1 template with oMTV663 & oMTV664)[b] and (3) vector DNA (pUC18 template with oMTV665 & oMTV666)[b] *SBF51961* junction	This study
pBAC1415	Ap^R; synthetic bridging fragment for ACIAD_RS07910 and ACIAD_RS07915= *pobS* (Site 1); NEBuilder assembly of three PCR-generated fragments: (1) ACIAD_RS07915=p*obS* (ADP1 template with oMTV655 & oMTV656)[b], (2) ACIAD_RS07910 (ADP1 template with oMTV657 & oMTV658)[b] and (3) vector DNA (pUC18 template with oMTV659 & oMTV660)[b] junction *SBF51959*	This study
pBAC1486	Ap^{R}Km^R; *praRABEGFDCHI*-ΩK*51899* with flanking ADP1 sequence for integration at Site 2 (ACIAD_RS07695, ACIAD_RS07700 and ACIAD_RS07705); with foreign DNA (*pra* genes ΩK) were inserted in the SacI site of pBAC1397. Genes are oriented as shown in Figure S	This study
pBAC1518	Ap^{R}Km^R; *praRABEGFDCHI*::ΩK*51936*; *praC51936*; *pra* genes inserted into pBAC1396 linearized with SpeI by gap repair with ACN1936	This study
pBAC1526	Ap^R; *pobR-hcaG*; NEBuilder assembly of a PCR-generated fragment, *pobR-hcaG* region (ADP1 template with oMTV752 & oMTV753)[b], assembled to vector backbone from BamHI-digested pUC18	This study
pBAC1539	Ap^{R}Km^R; *sacB*-ΩK from pRMJ1 digested with BamHI inserted in BamHI in pUC18	This study

pBAC1548	$Ap^RSm^RSp^R$; *sacB*-ΩS; ΩS from pUI1638 digested with Eco53KI inserted between Eco53KI and EcoRV of pBAC1539	This study
pBAC1561	Ap^R; Δ*pcaUIJFBDCHG*::P_{lac}-*pcaK52106*	[4]
pBAC1575	Ap^R; *pobA* region (*pobS-hcaC*); NEBuilder assembly of three PCR-generated fragments: (1) *pobS-pobA* (ACN1829 template with oMTV802 & oMTV803), (2) *hcaG-hcaC* (ADP1 template with oMTV804 & oMTV805), and (3) plasmid backbone (pUC18 template with oMTV813 & oMTV814)	This study
pBAC1578	Ap^R; Δ*pobA52440*; NEBuilder assembly of two PCR-generated fragments: (1) *pobS-pobR* (ADP1 template with oMTV802 & oMTV817)[b] and (2) *hcaG-hcaC* (ADP1 template with oMTV818 & oMTV805)[b] assembled to XbaI-digested pUC18	This study
pBAC1584	$Ap^RSm^RSp^R$; *pobA*::ΩS*52212*; ΩS from pUI1638 digested with SalI and ligated into pBAC1575 (XhoI digested)	This study
pBAC1585	$Ap^RSm^RSp^R$; *hcaE*::*sacB*-ΩS*52416*; *sacB*-ΩS from pBAC1548 was digested with XbaI and ligated to NbeI-digested pBAC1578	This study
pBAC1642	Ap^RKm^R; *praRABEGFDHI*::ΩK*52237*; NEBuilder assembly of two PCR-generated fragments: (1) *praGFD* (pBAC1413 template with oMTV836 & oMTV837)[b] and (2) *praHI* (pBAC1413 template with oMTV838 & oMTV839)[b] assembled to vector backbone from BsiWI-digested pBAC1413	This study
pBAC1643	Ap^RKm^R; *praRABEGFDCHI*::ΩK*52045*; *praC52045*; *pra* genes inserted into pBAC1396 linearized with SpeI by gap repair with ACN2045	This study
pBAC1674	Ap^R; *praRABEG*; pBAC1642 digested with SalI and fragment containing *praRABEG* was religated	This study
pBAC1702	Ap^R; *pcaK* and mCherry controlled by the D5H6 promoter replacing *pcaUIJFBDKCHG*	[4]
pBAC1711	Ap^R; *gacA52276*; PCR cloning of one PCR-generated fragment: (1) *pilR-pbpG* (ACN2317 template with oMTV918 & oMTV919) assembled to cloning vector pMiniT	This study
pBAC1713	Ap^R; *sacB*-ΩS; NEBuilder assembly of two PCR-generated fragments: (1) *sacB*-ΩS (pBAC1548 template with oMTV970 and oMTV971)[b] and (2) plasmid backbone (pUC18 template with oMTV968 and oMTV969)[b]	This study
pBAC1717	Ap^R; mCherry removed from pBAC1702 resulting in P_{D5H6}-*pcaK52401*	[4]
pBAC1783	Ap^RKm^R; *praRABEGFDH*-ΩK*52506*; constructed by *E. coli* assembly[c] of fragments made by PrimeSTAR PCR: (1) plasmid backbone and *praRABEGFDH* (pBAC1674 template with oMTV1108 & oMTV1109)[b], (2) Δ*praI* region (pBAC1642 template with oMTV1110 & oMTV1111)[b], and (3) ΩK (pBAC1413 template with oMTV1112 & oMTV1113)[b]	This study
pBAC1784	Ap^R; Δ*pobA*::*praI*; NEBuilder assembly of four PCR-generated fragments: (1) plasmid backbone (pUC18 template with oMTV1114 & oMTV1115)[b], (2) *pobR* (pBAC1578 template with oMTV1116 & oMTV1117)[b], (3) *praI* (pBAC1784 template with oMTV1118 & oMTV1119)[b], and (4) *hcaG* (pBAC1578 template with oMTV1120 & oMTV1121)[b]	This study

pBAC1786	$Ap^RSm^RSp^R$; *gacA*::*sacB*-ΩS*52474*; *sacB*-ΩS from pBAC1713 was digested with Eco53KI and ligated to StuI-digested pBAC1711	This study
pBAC1788	Ap^RKm^R; *praH-pobA*-ΩK-*pobS*; NEBuilder assembly of four PCR-generated fragments: (1) plasmid backbone (pUC18 template with oMTV1114 & oMTV1115)[b], (2) p*raH* (pBAC1642 template with oMTV1122 & oMTV1123)[b], (3) *pobA* (pBAC1575 template with oMTV1124 & oMTV1125)[b], and (4) ΩK-*pobS* (pBAC1413 template with oMTV1126 & oMTV1127)[b]	This study
pBAC2184	Ap^R; Δ*gacA53315*; constructed by *E. coli* assembly[c] of fragments made by PrimeSTAR PCR: (1) plasmid backbone (pUC18 template with oCDM24 & oCDM25)[b], (2) ACIAD_RS01215-ACIAD_RS01225 (ADP1 template with oCDM489 & oCDM490)[b], and (3) *pbpG*-ACIAD_RS01245 (ADP1 template with oCDM491 & oCDM492)[b]	This study
pBAC2207	Ap^R; carries two ADP1 DNA fragments on either side of an engineered deletion: 1) **1,005,604-1,007,093** and 2) **973,412-974,865** Corresponds to a 31 kbp chromosomal deletion between **974,866-1,005,605**; Δ31(A)-kbp, **Figure S4**	This study
pBAC2208	Ap^R; carries two ADP1 DNA fragments on either side of an engineered deletion: 1) **1,003,725-1,005,318** and 2) **1,035,393-1,037,038** Corresponds to a 31 kbp chromosomal deletion between **1,005,319-1,035,392;** Δ31(B)-kbp, **Figure S4**	This study
pBAC2217	Ap^R; carries two ADP1 DNA fragments on either side of an engineered deletion: 1) **962,260-964,808** and 2) **974,250-977,052** Corresponds to a 9 kbp chromosomal deletion between **964,809-974,249;** Δ9-kbp, **Figure S4**	This study
pBAC2218	Ap^R; carries two ADP1 DNA fragments on either side of an engineered deletion: 1) **942,730-944,857** and 2) **959,116-961,446** Corresponds to a 14 kbp chromosomal deletion between **944,858-959,115;** Δ14-kbp, **Figure S4**	This study
pBAC2209	Ap^R; ΔACIAD_RS04410-ACIAD_RS04550; constructed by *E. coli* assembly[c] of fragments made by PrimeSTAR PCR: (1) plasmid backbone (pUC19 template with oACB225 &oACB226)[b], (2) ACIAD_RS04405-ACIAD_RS04410 (ADP1 template with oACB227 & oACB228), and (3) ACIAD_RS04550-ACIAD_RS04560 (ADP1 template with oACB223 & oACB224)[b]	This study
pBAC2220	$Ap^RSm^RSp^R$; ΔACIAD_RS04410-ACIAD_RS04550*53419*:: ΩS; ΩS from pUI1638 digested with PstI ligated to PstI-digested pBAC2209	This study
pBAC2245	Ap^RGm^R; carries two ADP1 DNA fragments on either side of an engineered *vanK* deletion: 1) **963,236-968,382** and 2) **969,761-972,598**; *vanK* was replaced by a fragment with two convergent genes encoding Gm^R and *sacB*; Note: *vanP* downstream of *vanK* loses its RBS and promoter Corresponds to a chromosomal deletion of DNA: **968,383-969,760**	This study

[a]Abbreviations: Ampicillin (Ap), Streptomycin (Sm), Spectinomycin (Sp), Kanamycin (Km), omega cassette conferring Km^R (ΩK) or Sm^RSp^R (ΩS) [7]; splicing by overlap extension PCR (SOEing).[9]

[b]Oligos (primers) used for PCR are underlined and shown in Table **S3**.

[c]*E. coli* assembly refers to the method of Kostylev et al.[10] PCR products for this method were typically generated with PrimeSTAR polymerase.

[d]Bold numbers correspond to positions on the ADP1 chromosome in NCBI entry NC_005966.

Table S3. Primers

**Primer**	**Sequence (5'→3')**	**Use**
oACB223	GCTTTCTTGCGTCATAGCTGTTTCCTGTGTG	With oACB224 amplifies vector DNA from pUC19 to make pBAC2209
oACB224	TTAGTCTTGAACTGGCCGTCGTTTTACAAC	With oACB224 amplifies vector DNA from pUC19 to make pBAC2209
oACB225	GACGGCCAGTTCAAGACTAAGTGATTCCTC	With oACB226 amplifies ACIAD_RS04405-ACIAD_RS04410 from ADP1 to make pBAC2209
oACB226	TTTCTAGGTGAACTCACACGAGCCTATAG	With oACB225 amplifies ACIAD_RS04405-ACIAD_RS04410 from ADP1 to make pBAC2209
oACB227	CGTGTGAGTTCACCTAGAAAAATTGCATTGGGATG	With oACB228 amplifies ACIAD_RS04550-ACIAD_RS04560 from ADP1 to make pBAC2209
oACB228	CAGCTATGACGCAAGAAAGCGCCACGATTG	With oACB227 amplifies ACIAD_RS04550-ACIAD_RS04560 from ADP1 to make pBAC2209
oCDM24	CTGCAGGTCGACTCTAGAGGATCCC	With oCDM25 amplifies vector DNA from pUC18 to make pBAC2184
oCDM25	GCATGCAAGCTTGGCACTGGCCG	With oCDM24 amplifies vector DNA from pUC18 to make pBAC2184
oCDM489	GATCCTCTAGAGTCGACCTGCAGCATGAAAGATGAGACTTATTTGGAAAAT	With oCDM490 amplifies ACIAD_RS01215-ACIAD_RS01225 from ADP1 to make pBAC2184
oCDM490	GAGGCCTGTGTGTAATCTGCGCTACAAGGATGATTG	With oCDM489 amplifies ACIAD_RS01215-ACIAD_RS01225 from ADP1 to make pBAC2184
oCDM491	TAGCGCAGATTACACACAGGCCTCCTGAAATTACAAAG	With oCDM492 amplifies *pbpG*-ACIAD_RS01245 from ADP1 to make pBAC2184
oCDM492	CGGCCAGTGCCAAGCTTGCATGCCGAATTACGCGTACATCCAACCC	With oCDM491 amplifies *pbpG*-ACIAD_RS01245 from ADP1 to make pBAC2184

DOI: 10.1021/acssynbio.5c00341

oMTV274	GCTCGACGCCTTCTATTTCAA	With oMTV275, used in qPCR to detect *rpoA* copy number
oMTV275	TTTACGTCGCATTCTATTGTCTTCTT	With oMTV274, used in qPCR to detect *rpoA* copy number -
oMTV568	CCATGATTACGAATTCATGACACCAGCTTGCAAATTAC	With oMTV609, amplifies ACIAD_RS07700 from ADP1 to make pBAC1397
oMTV569	TTGCATGCCTGCAGTTAGCCACAGTTCAAATGTGCTT	With oMTV608, amplifies ACIAD_RS07705 from ADP1 to make pBAC1397
oMTV570	CATTTGAACTGTGGCTAACTGCAGGCATGCAAGCTT	With oMTV571, amplifies vector DNA from pUC18 to make pBAC1397
oMTV571	TGCAAGCTGGTGTCATGAATTCGTAATCATGG	With oMTV570, amplifies vector DNA from pUC18 to make pBAC1397
oMTV592	GTCTGATTACCAATTTGCGGCA	With oMTV669, amplifies novel junction DNA that results from amplification of DNA at Site 1
oMTV608	CAGTGATTAGATAGTTGAGCTCTTAGTTAATGCCATAGCACTGCTT	With oMTV569, amplifies ACIAD_RS07705 from ADP1 to make pBAC1397; **SacI** site introduced
oMTV609	CTATGGCATTAACTAAGAGCTCAACTATCTAATCACTGAAATTTTTATTC	With oMTV568, amplifies ACIAD_RS07700 from ADP1 to make pBAC1397; **SacI** site introduced
oMTV610	GAATTGGAGCTCCACTAGTAATACCCCCAAGAATGACAG	With oMTV611 amplifies *praRA* from pBAC1384 to make pBAC1406; **SpeI** site introduced
oMTV611	TGACCATGATTACGCCAAGGGCCCAAAAGCTGG	With oMTV610 amplifies *praRA* from pBAC1384 to make pBAC1406; **PspOMI** site introduced
oMTV612	CCAGCTTTTGGGCCCTTGGCGTAATCATGGTCA	With oMTV613 amplifies vector backbone from pBAC1384 to make pBAC1406; **PspOMI** site introduced
oMTV613	ATTCTTGGGGGTATTACTAGTGGAGCTCCAATTCGCCC	With oMTV612 amplifies vector backbone from pBAC1384 to make pBAC1406; **SpeI** site introduced
oMTV655	CCATGATTACGAATTCGCCTAATCTATAAGCGGAAGTTGC	With oMTV656 amplifies ACIADRS07915 from ADP1 to make pBAC1415
oMTV656	TTTACCTGTCACACGGGAATCGGAGTGGACAGTTTT	With oMTV655 amplifies ACIADRS07915 from ADP1 to make pBAC1415

DOI: 10.1021/acssynbio.5c00341

oMTV657	TCCACTCCGATTCCCGTGTGACAGGTAAACCACT	With oMTV668 amplifies ACIADRS07910 from ADP1 to make pBAC1415
oMTV658	TGCATGCCTGCAGCAAGGGCATATGCAATCACGTA	With oMTV667 amplifies ACIADRS07910 from ADP1 to make pBAC1415
oMTV659	TTGCATATGCCCTTGCTGCAGGCATGCAAG	With oMTV660 amplifies vector DNA from pUC18 to make pBAC1415
oMTV660	TCCGCTTATAGATTAGGCGAATTCGTAATCATGG	With oMTV659 amplifies vector DNA from pUC18 to make pBAC1415
oMTV661	CCATGATTACGAATTCATGTATACCTGAGCACGCC	With oMTV662 amplifies ACIAD_RS07705 from ADP1 to make pBAC1414
oMTV662	TTGAATTAATTTGGCTCTCAAATGTGCTTGTTGATCCAAA	With oMTV661 amplifies ACIAD_RS07705 from ADP1 to make pBAC1414
oMTV663	AACAAGCACATTTGAGAGCCAAATTAATTCAACGCTT	With oMTV664 amplifies ACIAD_RS07700 from ADP1 to make pBAC1414
oMTV664	TGCATGCCTGCAGTTAGTTAATGCCATAGCACTGCTT	With oMTV663 amplifies ACIAD_RS07700 from ADP1 to make pBAC1414
oMTV665	GCTATGGCATTAACTAACTGCAGGCATGCA	With oMTV666 amplifies vector DNA from pUC18 to make pBAC1414
oMTV666	TGCTCAGGTATACATGAATTCGTAATCATGG	With oMTV665 amplifies vector DNA from pUC18 to make pBAC1414
oMTV667	ATGGTATGCCTCGTTATTGGGTC	With oMTV668, amplifies novel junction DNA that results from amplification of DNA at Site 2
oMTV668	CAGTGATTTGGGTGCCATTTG	With oMTV667, amplifies novel junction DNA that results from amplification of DNA at Site 2
oMTV669	GCACGAGCAAGGTAATGATG	With oMTV592, amplifies novel junction DNA that results from amplification of DNA at Site 1
oMTV680	AGCGGGCGGTGTTCATC	With oMTV681, used in qPCR to detect F52 copy number
oMTV681	CCCCGGACCAAGTTGTGA	With oMTV680, used in qPCR to detect F52 copy number
oMTV752	ATTCGAGCTCGGTACCCGGGTTATACCAAATTACGCAGCTC	With oMTV753 amplifies *pobR-hcaG* from ADP1 to make pBAC1526

oMTV753	CCTGCAGGTCGACTCTAGAGGTACGATAAACTGATGGCAAAATAC	With oMTV752 amplifies *pobR-hcaG* from ADP1 to make pBAC1526
oMTV802	AATTCGAGCTCGGTACCCGGGGCCTCTTCGGAATCGTTTTCCG	With oMTV817 amplifies *pobS-pobR* from ADP1 to make pBAC1578. With oMTV803 amplifies *pobS-pobR* from ADP1 to make pBAC1575
oMTV803	CACTTGTAAATAAACACAGCCGAGATAAAAAAG	With oMTV802 amplifies *pobS-pobA* from ACN1829 template
oMTV804	ATCTCGGCTGTGTTTATTTACAAGTGAAATTCTCG	With oMTV805 amplifies *hcaG-hcaC* from ADP1 to make pBAC1575
oMTV805	AAGCTTGCATGCCTGCAGGTCGACTATGAATGCCACTCAATCAC	With oMTV818 amplifies *hcaG-hcaC* from ADP1 to make pBAC1578. With oMTV804 amplifies *hcaG-hcaC* from ADP1 to make pBAC1575
oMTV813	ATTGAGTGGCATTCATAGTCGACCTGCAGG	With oMTV814 amplifies vector backbone from pUC18 to make pBAC1575
oMTV814	ATTCCGAAGAGGCCCCGGGTACCG	With oMTV813 amplifies vector backbone from pUC18 to make pBAC1575
oMTV817	TCTTTTTGTTAACATCATCCTTGCTATTTTCTATTTTTA	With oMTV802 amplifies *pobS-pobR* from ADP1 template to make pBAC1578
oMTV818	GGATGATGTTAACAAAAAGAGAGCGATTAG	With oMTV805 amplifies *hcaG-hcaC* from ADP1 to make pBAC1578
oMTV836	AAGAGATGGTTGCCTCCATTCAATCGTACGTGCCGGGCTACCG	With oMTV837 amplifies *praGFD* from pBAC1413 to make pBAC1642
oMTV837	CCTCATGACCTCCCTATTCTTTGACAATAAAATCGATCGTACC	With oMTV836 amplifies *praGFD* from pBAC1413 to make pBAC1642
oMTV838	TGTCAAAGAATAGGGAGGTCATGAGGATGTAC	With oMTV839 amplifies *praHI* from pBAC1413 to make pBAC1642
oMTV839	GATCGTTCCCTCGATCTCCTCACGCGTACGGTTCTCGATAATAATG	With oMTV838 amplifies *praHI* from pBAC1413 to make pBAC1642
oMTV918	AGTCAGTGAGCGAGGAAGCGGAAGATTATTCGTTTTCCTTTATTGAGCACTG	With oMTV919 amplifies *pilR-pbpG* from ACN2317 to make pBAC1711
oMTV919	GAGAGGCGGTTTGCGTATTGGGCGCTTAAATTCGTTTTGGTTGTTGCGCTAAC	With oMTV918 amplifies *pilR-pbpG* from ACN2317 to make pBAC1711
oMTV968	TCGTAATCATGGTCATAGCTG	With oMTV969 amplifies vector DNA from pUC18 to make pBAC1713
oMTV969	ATTCGAGCTCGGTACCCG	With oMTV968 amplifies vector DNA from pUC18 to make pBAC1713

oMT970	AGCTATGACCATGATTACGAGAGCTCCACCGCGGTGGC	With oMTV971 amplifies *sacB*-ΩS from pBAC1548 to make pBAC1713. **Eco53KI** site introduced
oMT971	CCCGGGTACCGAGCTCGAATAAGCTTGCATGCCTGCAGGTC	With oMTV970 amplifies *sacB*-ΩS from pBAC1548 to make pBAC1713. **Eco53KI** site introduced
oMTV1108	CTAACGTATAGACCGTATCGCG	With oMTV1109 amplifies *praRABEG* and vector backbone from pBAC1674 to make pBAC1783
oMTV1109	TCGACGGATCAGTGAGGGTTTG	With oMTV1108 amplifies *praRABEG* and vector backbone from pBAC1674 to make pBAC1783
oMTV1110	GCGCGATACGGTCTATACGTTAGTCGACCCCGACAAGATGGAC	With oMTV1111 amplifies *praGFDH* from pBAC1642 to make pBAC1783
oMTV1111	AGCCCACTTCCGACATACCTCCTGGAGCTCTTAAGGG	With oMTV1110 amplifies *praGFDH* from pBAC1642 to make pBAC1783
oMTV1112	CAGGAGGTATGTCGGAAGTGGGCTGCAGGAATTC	With oMTV1113 amplifies ΩK from pBAC1413 to make pBAC1783
oMTV1113	GCAAACCCTCACTGATCCGTCGACCAAAGCGGCCATCGTGCCT	With oMTV1112 amplifies ΩK from pBAC1413 to make pBAC1783
oMTV1114	GACTCTAGAGGATCCCCGGG	With oMTV1115 amplifies backbone from pUC18 to make pBAC1784 or pBAC1788
oMTV1115	GACCTGCAGGCATGCAAGCTTG	With oMTV1114 amplifies backbone from pUC18 to make pBAC1784 or pBAC1788
oMTV1116	GTACCCGGGGATCCTCTAGAGTCTTATACCAAATTACGCAGCTC	With oMTV1117 amplifies *pobR* from pBAC1578 to make pBAC1784
oMTV1117	CTGAGTTCGCATAACATCATCCTTGCTATTTTC	With oMTV1116 amplifies *pobR* from pBAC1578 to make pBAC1784
oMTV1118	AAGGATGATGTTATGCGAACTCAGGTTGGCATTATTG	With oMTV1119 amplifies *praI* from pBAC1413 to make pBAC1784
oMTV1119	TCTCTTTTTGTTCTAAAATTCCATTGGCAGTCCG	With oMTV1118 amplifies *praI* from pBAC1413 to make pBAC1784
oMTV1120	ATGGAATTTTAGAACAAAAAGAGAGCGATTAGTC	With oMTV1121 amplifies *hcaG* from pBAC1578 to make pBAC1784
oMTV1121	CCAAGCTTGCATGCCTGCAGGTCATGAAAAAACCTCAACATTTTTTTAAA C	With oMTV1120 amplifies *hcaG* from pBAC1578 to make pBAC1784
oMTV1122	GTACCCGGGGATCCTCTAGAGTCATGTACGATGTTCATACGCATTTTATC	With oMTV1123 amplifies *praH* from pBAC1642 to make pBAC1788

DOI: 10.1021/acssynbio.5c00341

oMTV1123	CATAGTTTGCATACATACCTCCTGGAGCTCTTAAG	With oMTV1122 amplifies *praH* from pBAC1642 to make pBAC1788
oMTV1124	CAGGAGGTATGTATGCAAACTATGAAAACCAAAG	With oMTV1125 amplifies *pobA* from pBAC1575 to make pBAC1788
oMTV1125	AGCCCACTTCCGTTAGCTGGCATGTTTTAAATAG	With oMTV1124 amplifies *pobA* from pBAC1575 to make pBAC1788
oMTV1126	CATGCCAGCTAACGGAAGTGGGCTGCAGGAAT	With oMTV1127 amplifies ΩK from pBAC1413 to make pBAC1788
oMTV1127	CCAAGCTTGCATGCCTGCAGGTCCTACAATACATCTCAATCTTTAATTATCAACTGTGAAG	With oMTV1126 amplifies ΩK from pBAC1413 to make pBAC1788

Table S4. Mutations in ACN1936 ($4HB^+$) compared to its parent strain, ACN1829 ($4HB^-$)

**Predicted gene or locus tag where mutations were identified**<br><br>**Position and direction of CDS**[a]	**ADP1 genome position and type of mutation**[b]	**Amino acid change (total residues in WT product)**<br>**Effect of mutation (total residues in altered gene product)**	**Predicted function of gene product**
ACIAD_RS01270<br>269,688→269,939	269,831<br>1-bp deletion	$K_{48} \rightarrow N_{48}$ (83 AA)<br>Frame shift (49 AA)	Hypothetical protein<br>DUF6587 family protein
rpoB<br>302,457→ 306,545	306,285<br>Point mutation<br>C → T	$P_{1277} \rightarrow S_{1277}$ (1362 AA)<br>AA replacement (1362 AA)	DNA-directed RNA polymerase subunit beta
pbpA (also known as *mrdA*)<br>1,090,713 ← 1,109,550	1,091,707-1,091,710<br>1-bp insertion<br>GGGG→GGGGG in CDS	$G_{345} \rightarrow G_{345}$ (675 AA)<br>Frameshift (349 AA)	penicillin-binding protein 2
ACIAD_RS06205 ←/←<br>ACIAD_RS06210<br>Intergenic region 1,341,597-1,341,802	1,341,621<br>Point mutation<br>G → A	N/A; Within 206 bp intergenic region; 25 bp upstream of CDS for superoxide dismutase (ACIAD_RS06205)	
praC<br>192 bp CDS	Nonnative *pra*-genes in Site 1; 1-bp insertion after nt 174 of CDS<br>AT → AAT	$S_{59} \rightarrow I_{59}$ (63 AA)<br>Frame shift (74 AA)	4-oxalocrotonate tautomerase
pobS – hcaC<br>1,723,656 ← 1,733,844	1,723,735 – 1,732,743<br>9,009-bp deletion[c]<br><br>See image below	Deletion of genes involved in the catabolism of 4HB and hydroxycinnamates (*pob* and *hca* genes, respectively)	PobS Putative regulator<br>PobR Regulator<br>PobA 4HB hydroxylase<br>HcaG Esterase<br>HcaF Thioesterase<br>HcaE Porin<br>HcaD Acyl-CoA dehydrogenase<br>HcaC CoA ligase

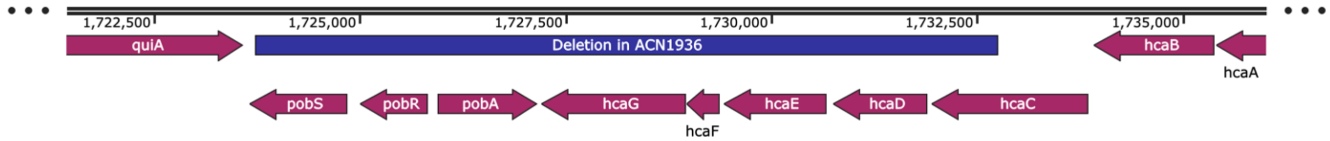

[a]Arrows in left column indicate direction of genes and of mutations in intergenic regions: e.g. ←/→ for mutations between divergent coding sequences

[b]Numbers correspond to positions on the ADP1 chromosome in NCBI entry NC_005966.

[c]9,009-bp deletion includes complete deletion of the following genes (and predicted protein product): ACIAD_RS07920 (PobR, an IclR family transcriptional regulator), *pobA* (4-hydroxybenzoate 3-monooxygenase), ACIAD_RS07930 (tannase/feruloyl esterase family alpha/beta hydrolase), ACIAD_RS07935 (PaaI family thioesterase), ACIAD_RS07940 (OprD family porpin), ACIAD_RS07945

(acyl-CoA dehydrogenase family protein) and partial deletion of: *pobS*, ACIAD_RS07915 (tetratricopeptide repeat protein) and ACIAD_RS07950 (feruloyl-CoA synthase)

Table S5. Mutations in *praC*, encoding 4-oxalocrotonate tautomerase

Allele designation and origin	Position of mutation in the 192 bp coding sequence (CDS) of the *praC* gene	Amino acid change (total residues in PraC)<br>Effect of mutation (total residues in altered gene product)
praC51936<br><br>First identified in EASy derived $4HB^+$ strain ACN1936	1-bp insertion after nt 174 of CDS<br>AT → AAT	S_{59} → I_{59} (63 AA)<br>Frame shift (74 AA)
praC52608<br><br>First identified in EASy derived $4HB^+$ strain ACN2608	8-bp deletion after nt 92 of CDS<br>GTT ACC → GTT GGT	T_{25} → G_{25} (63 AA)<br>Frame shift (61 AA)
praC52611<br><br>First identified in EASy derived $4HB^+$ strain ACN2611	2-bp deletion after nt 151 of CDS<br>GGGG → GG	G_{52} → N_{52} (63 AA)<br>Frame shift (73 AA)
Δ*praC52237*<br><br>Engineered on plasmid pBAC1642	*praC* CDS removed<br><br>783 bp deleted immediately after the stop signal (TAG) of *praD.* The RBS for *praH* is intact	N/A
praC53315<br><br>First identified in CEMENT derived $4HB^+$ strain ACN3315	1-bp insertion after nt 173 of CDS<br>GCA TCG → GCC ATC	S_{59} → I_{59} (63 AA)<br>Frame shift (74 AA)

DOI: 10.1021/acssynbio.5c00341

Table S6. Mutations in 4HB⁺ strains derived from a recipient strain (ACN1829) transformed by different combinations of donor DNA in CEMENT assays

	Donor DNA used to transform the 4HB⁻ recipient[a]			
	ΔgacA<br>*ΔpraC*<br>*ΔpobA*	*ΔgacA*<br>*ΔpraC*	*ΔgacA*<br>*ΔpobA*	*ΔpraC*<br>*ΔpobA*
4HB⁺ Transformant[b]	ACN3316	ACN3317	ACN3315	ACN3318
Chromosomal incorporation of all donor alleles?	Yes	Yes	Yes	Yes
Additional mutated genes	None	None	Two:	Two:
			praC<br>1 bp insertion after nt 173 of CDS GCA TCG → GCC ATC, causes frame shift<br>S_{59} → I_{59} (encodes 74 AA variant, rather than 63 AA wild-type protein)<br><br>***relA***<br>**2,992,794 ← 2,995,100**<br>Point mutation (A→C, **2,993,108)**<br>Y_{665} → D_{665}, AA replacement in 768 AA RelA (GTP pyrophos-phokinase)	***gacS***<br>**2,998,466 → 3,001,264**<br>1-bp deletion at position 3,000,342<br>Frameshift affects protein sequence R_{626} → L_{626} (encodes 633 AA variant, rather than 932 AA)<br>Frame shift (633 AA wild-type protein)<br><br>***benK***<br>**1,431,562 → 1,432,962**<br>Point mutation (G→A, **1,432,778)**<br>W_{406} → Stop truncates the protein after 405 AA, instead of encoding the WT 466 AA BenK (benzoate transporter)

[a]Linear DNA fragments carrying the engineered alleles were obtained by restriction digestion of the following plasmids: pBAC2184 (Δ*gacA53315*), pBAC1578 (Δ*pobA52440),* and pBAC1642 (Δ*praC52237*).

[b]Transformants arising on the 4HB plate of a CEMENT assay were streak purified, named, and characterized further by whole genome sequencing

REFERENCES

(1) Vaneechoutte, M.; Young, D. M.; Ornston, L. N.; De Baere, T.; Nemec, A.; Van Der Reijden, T.; Carr, E.; Tjernberg, I.; Dijkshoorn, L. Naturally transformable *Acinetobacter* sp. strain ADP1 belongs to the newly described species *Acinetobacter baylyi*. *Appl Environ Microbiol* **2006**, *72* (1), 932-936 DOI: 10.1128/AEM.72.1.932-936.2006.

(2) Singh, A.; Bedore, S. R.; Sharma, N. K.; Lee, S. A.; Eiteman, M. A.; Neidle, E. L. Removal of aromatic inhibitors produced from lignocellulosic hydrolysates by *Acinetobacter baylyi* ADP1 with formation of ethanol by *Kluyveromyces marxianus*. *Biotechnol Biofuels* **2019**, *12*, 91. DOI: 10.1186/s13068-019-1434-7.

(3) Kasai, D.; Fujinami, T.; Abe, T.; Mase, K.; Katayama, Y.; Fukuda, M.; Masai, E. Uncovering the protocatechuate 2,3-cleavage pathway genes. *J Bacteriol* **2009**, *191* (21), 6758-6768. DOI: 10.1128/JB.00840-09.

(4) Biggs, B. W.; Bedore, S. R.; Arvay, E.; Huang, S.; Subramanian, H.; McIntyre, E. A.; Duscent-Maitland, C. V.; Neidle, E. L.; Tyo, K. E. J. Development of a genetic toolset for the highly engineerable and metabolically versatile *Acinetobacter baylyi* ADP1. *Nucleic Acids Res* **2020**, *48* (9), 5169-5182. DOI: 10.1093/nar/gkaa167.

(5) Jones, R. M.; Williams, P. A. Mutational analysis of the critical bases involved in activation of the AreR-regulated sigma54-dependent Promoter in *Acinetobacter* sp. Strain ADP1. *Appl Environ Microbiol* **2003**, *69* (9), 5627-5635. DOI: 10.1128/AEM.69.9.5627-5635.2003.

(6) Norrander, J.; Kempe, T.; Messing, J. Construction of improved M13 vectors using oligodeoxynucleotide-directed mutagenesis. *Gene* **1983**, *26* (1), 101-106. DOI: 10.1016/0378-1119(83)90040-9.

(7) Eraso, J. M.; Kaplan, S. *prrA*, a putative response regulator involved in oxygen regulation of photosynthesis gene expression in *Rhodobacter sphaeroides*. *J Bacteriol* **1994**, *176* (1), 32-43. DOI: 10.1128/jb.176.1.32-43.

(8) Tumen-Velasquez, M.; Johnson, C. W.; Ahmed, A.; Dominick, G.; Fulk, E. M.; Khanna, P.; Lee, S. A.; Schmidt, A. L.; Linger, J. G.; Eiteman, M. A.; et al. Accelerating pathway evolution by increasing the gene dosage of chromosomal segments. *Proc Natl Acad Sci U S A* **2018**, *115* (27), 7105-7110. DOI: 10.1073/pnas.1803745115.

(9) Horton, R. M.; Cai, Z. L.; Ho, S. N.; Pease, L. R. Gene splicing by overlap extension: tailor-made genes using the polymerase chain reaction. *BioTechniques* **2013**, *54* (3), 129-133. DOI: 10.2144/000114017.

(10) Kostylev, M.; Otwell, A. E.; Richardson, R. E.; Suzuki, Y. Cloning should be simple: *Escherichia coli* DH5 alpha-mediated assembly of multiple DNA fragments with short end homologies. *Plos One* **2015**, *10* (9). DOI: 10.1371/journal.pone.0137466.
